# Leigh Syndrome: A Comprehensive Review of the Disease and Present and Future Treatments

**DOI:** 10.3390/biomedicines13030733

**Published:** 2025-03-17

**Authors:** Giuseppe Magro, Vincenzo Laterza, Federico Tosto

**Affiliations:** 1 Department of Neuroscience, “Giovanni Paolo II” Hospital, 88100 Lamezia Terme, Italy; 2 Department of Medical and Surgical Sciences, Institute of Neurology, Magna Graecia University, 88100 Catanzaro, Italy

**Keywords:** Leigh syndrome, genetics, neurology, therapy, preclinical research

## Abstract

Leigh syndrome (LS) is a severe neurodegenerative condition with an early onset, typically during early childhood or infancy. The disorder exhibits substantial clinical and genetic diversity. From a clinical standpoint, Leigh syndrome showcases a broad range of irregularities, ranging from severe neurological issues to minimal or no discernible abnormalities. The central nervous system is most affected, resulting in psychomotor retardation, seizures, nystagmus, ophthalmoparesis, optic atrophy, ataxia, dystonia, or respiratory failure. Some patients also experience involvement of the peripheral nervous system, such as polyneuropathy or myopathy, as well as non-neurological anomalies, such as diabetes, short stature, hypertrichosis, cardiomyopathy, anemia, renal failure, vomiting, or diarrhea (Leigh-like syndrome). Mutations associated with Leigh syndrome impact genes in both the mitochondrial and nuclear genomes. Presently, LS remains without a cure and shows limited response to various treatments, although certain case reports suggest potential improvement with supplements. Ongoing preclinical studies are actively exploring new treatment approaches. This review comprehensively outlines the genetic underpinnings of LS, its current treatment methods, and preclinical investigations, with a particular focus on treatment.

## 1. Introduction

### 1.1. Brief History

Leigh syndrome (LS) is a rare incurable mitochondrial disease with an early onset, typically during early childhood or infancy. Prof. Denis Leigh gave a very clear definition of subacute necrotizing encephalomyelopathy in 1951. He described a neuropathological entity characterized by early-onset development of necrotizing lesions in the grey nuclei of the brainstem, subthalamic region, and basal ganglia, including often the deep structures of the cerebellum and descending up to the rostral segment of the spinal cord. These lesions are usually symmetric, with neuronal loss, initial inflammation followed by gliosis, and maintenance of capillary hyperproliferation. Any Leigh syndrome or Leigh-like syndrome (whose definition is much more uncertain) must contain these essential neuropathological features [1].

### 1.2. Most Common Clinical Presentation

The symptoms are various: when the long tracts (e.g., the mesencephalic anterior descending tracts) are also affected, a motor impairment with axial hypotonia and spastic tetraparesis can occur. In all cases, movement disorders, due to lesions in the basal ganglia and subthalamus, are present, as well as respiratory irregularities (alterations of the respiratory centers), eye movement disorders (due to medial longitudinal fasciculus and oculomotor nuclei involvement), ataxia (cerebellum and spinocerebellar tracts), sometimes hypoacusia, and sometimes optic atrophy (retinitis pigmentosa is rather rare and more often detected in the m.8993T>G mutation [2]). A general psychomotor regression and cognitive delay or stagnation may occur. Characteristically, there are no consistent dysmorphic or malformation features, whereas the presence of “disease-free” windows typically lasts variably from 0 to 6 months. The first or second infection of the child usually precipitates the situation, revealing the general neurological impairment. The heart is usually unaffected; myopathy is not prominent, and ragged red fibers are usually absent. Peripheral neuropathy may be present, but it is not the main problem. Other organs are marginally or not affected at all, although a DeToni–Debré–Fanconi proximal tubular impairment may occasionally be seen [3]. Usually, serum lactate is relatively high, and sometimes, especially during infection, metabolic acidosis may occur. The natural history is that of progressive, rapid disease, rarely do these patients live beyond the first decade. Adult or late-onset conditions are rare and incompletely understood but are certainly documented [4]. Biochemically, most of the cases are associated with a mitochondrial bioenergetic disorder, either for mutations in nuclear DNA (nDNA) or mitochondrial DNA (mtDNA) (e.g., in the high heteroplasmic levels of the 8993T>G mutation of ATPase6 in mitochondrial inherited Leigh syndrome [5]). In several cases, Complex I (CI) mutations, either in structural subunits or assembly factors, are associated with Leigh syndrome, but the single most frequent gene causing nDNA Leigh syndrome is loss of function mutations in *SURF1*, an assembly factor of cytochrome c oxidase IV [4].

Leigh syndrome is defined in the Online Mendelian Inheritance in Man Database (ONIM 2014) as follows: (1) a neurodegenerative disease with variable symptoms (2) caused by mitochondrial dysfunction from a hereditary genetic defect, and (3) accompanied by bilateral central nervous system (CNS) lesions. These criteria are not always met, and, in those cases, the term “Leigh-like syndrome” is preferred [6,7]. Leigh and Leigh-like syndromes have defects in the respiratory chain, coenzyme Q, or pyruvate dehydrogenase complex [8]. The absence of well-defined criteria for LS results from the broadest genetic heterogeneity; in fact, non-mitochondrial disorders also may present as LS [9]. LS often shows a progressive decline of CNS because of focal necrotizing lesions of the basal ganglia, cerebellum, diencephalon, or brainstem, as already mentioned [10]. The estimated incidence is 1 per 40,000 live births [11]. Nonetheless, the precise prevalence of Leigh syndrome is not known, as many cases are wrongly diagnosed [12]. LS is a pan-ethnic disorder, yet some genetic subtypes have a high prevalence in certain geographical regions, as discussed in the following sections.

### 1.3. Faroe Islands Variant

A Faroe variant has been described in the Faroe Islands, where the incidence is higher due to a founder effect and a carrier frequency of one in thirty-three [11]. The term Faroe variant of mitochondrial disease has historically been used to describe a specific form of *SUCLA2* deficiency, a genetic disorder affecting succinate-CoA ligase, an essential enzyme in the tricarboxylic acid (TCA) cycle. The name derives from the increased prevalence of this condition in the Faroe Islands, attributed to a founder effect. However, the term “Faroe variant” is misleading, as *SUCLA2* mutations have been identified in multiple populations worldwide. This genetic variation is associated with the early onset of muscle weakness (hypotonia), muscle atrophy, compromised motor skills, hearing problems, seizures, and neuroimaging findings that point to Leigh syndrome [13]. Individuals with this variation often experience recurring respiratory tract infections. Many of these patients exhibit abnormal muscle tone (dystonia) or excessive involuntary movements (hyperkinesia), which may manifest as athetoid or choreiform movements [13]. Scoliosis is common in most of these patients, often requiring a supportive corset [13].

### 1.4. French-Canadian Variant

The clinical profile of individuals affected by the French-Canadian variant, also known as the Saguenay variant, includes developmental delay, moderate muscle weakness (hypotonia), limited facial and limb movements, unsteady movement of the trunk (truncal ataxia), a cautious wide-based gait, and intention tremor. These patients do not display cardiomyopathy, glycosuria, and aminoaciduria, which are frequently present in LS [14]. The majority of patients with this variant present severe lactic acidosis, hepatic steatosis, or a neurodegenerative disorder [14]. Unfortunately, most of these individuals succumb to a rapid and severe acidic crisis before reaching five years of age [15]. This condition is predominantly observed in the Saguenay–Lac-Saint-Jean region of Quebec, Canada, due to a founder effect in the population. It is caused by mutations in the *LRPPRC* (leucine-rich pentatricopeptide repeat-containing protein) gene, which encodes a protein critical for the transcriptional and translational regulation of mitochondrial DNA [16]. This dysfunction leads to severe mitochondrial impairment, particularly affecting the brain, liver, and other high-energy-demanding tissues. The *LRPPRC* plays a pivotal role in mitochondrial gene expression. It functions in stabilizing mitochondrial mRNA, regulating oxidative phosphorylation (OXPHOS), and maintaining mitochondrial homeostasis [17,18].

### 1.5. Objective

The objective of this review is to provide a clinical presentation of the different Leigh and Leigh-like syndromes with a particular focus on current treatment and future directions.

## 2. Search Strategy

We performed a comprehensive search in the online database PubMed. We did not exert any limitation in terms of language or time of publication. We used search terms such as Leigh syndrome, Leigh disease, subacute necrotizing encephalomyelopathies, and juvenile Leigh disease in combination with treatment. Reference lists of retrieved articles and relevant reviews were also manually searched. More than 1331 results were included in the literature research, conducted until 15 February 2025. Retrieved studies were revised and included based on the objective of the review. Unpublished data were not included.

## 3. Clinical Presentation

The original case described by Dr. Leigh in 1951 was that of a 7-month-old boy who developed rapid progressive neurological regression with subsequent death [19]. The child had a normal birth and development until he suddenly developed lethargy with feeding difficulty at 5 months after exposure to a viral illness. Post-mortem examination demonstrated bilateral symmetrical lesions of necrotic nature in the brainstem, thalami, and spinal cord [19]. The initial characterization of the disease was thought to be limited to the central nervous system (CNS) and peripheral nervous system (PNS) involvement; it is now known that non-neurologic manifestations also happen in LS, especially in Leigh-like syndrome. The age of onset is infancy or early childhood, but it can also occur rarely in adolescents and adults [20]. A total of 75% of patients show signs and symptoms of the disease by 1 month of age [21]. Presenting symptoms of LS include sudden death, developmental delays, and regression of neurological abilities [22,23]. LS is considered a mitochondrial disease because it impairs mitochondrial oxidative phosphorylation, which results in reduced ATP production and a consequent increase in glycolysis [24]. This can influence cells during development and may be the reason why developmental delay is the most common feature of LS, as shown in a meta-analysis of the clinical manifestation of LS, where developmental delay was reported as the most common clinical sign in 57% of patients [25]. Developmental delay was followed by respiratory dysfunction (34%), epileptic seizures (33%), poor feeding (29%), and weakness (27%) [25]. A hallmark of LS is the occurrence of acute neurological and, at times, systemic events called “decompensations” linked to psychomotor delay or regression, resulting in the loss of previously acquired skills [26].

**Figure 1:**
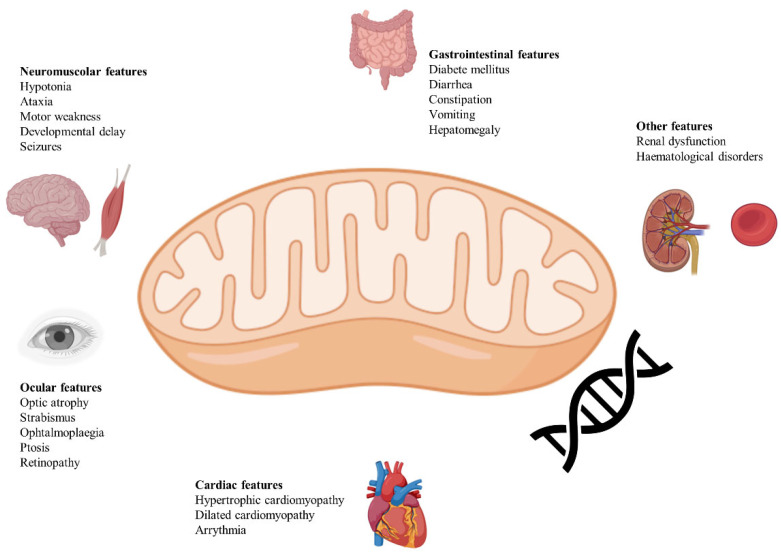
Clinical characteristics of Leigh syndrome. Predominantly impacting the brain, muscles, and eyes, the most common clinical features are highlighted. Additionally, abnormalities extend to the cardiovascular, gastrointestinal, renal, and hematological systems. The origin of Leigh syndrome lies in pathogenic mutations found in either nuclear DNA (nDNA) or mitochondrial DNA (mtDNA), leading to disruptions in the oxidative phosphorylation (OXPHOS) capabilities of the mitochondria. Mitochondria are represented at the center of the image, surrounded by clinical manifestations, despite nuclear gene mutations being more frequently responsible. This is due to Leigh syndrome being classified as a mitochondrial disease because it impairs mitochondrial oxidative phosphorylation, leading to reduced ATP production and a consequent increase in glycolysis.

Figure 1 provides a summary of neurological and non-neurological clinical manifestations of LS.

### 3.1. Neurological Manifestations

Most of the patients present with neurological manifestations of both the central and peripheral nervous systems, without other organ involvements [27]. Central nervous system manifestations include psychomotor retardation, optic atrophy, ataxia, nystagmus, ophthalmoparesis, dysphagia, cranial nerve palsies, general weakness, hypotonia, dystonia, deafness, and retinitis pigmentosa [28,29]. Respiratory manifestations can be classified as neurological manifestations too since they are often driven by brainstem involvement and/or severe myopathy. Acute respiratory failure is a frequent feature of Leigh syndrome, and it may happen without prodromal manifestations. When prodromal manifestations are present, they include irregular breathing, hyperventilation, hiccups, and lethargy [13,30,31]. Epileptic seizures are frequently observed in LS, with a reported prevalence between 40% and 79%. The types of seizures can vary, including generalized tonic-clonic, myoclonic, and focal seizures. Seizure occurrence is often associated with episodes of metabolic decompensation [7,32]. Focal seizures seem to be the most common type in a work from South Korea [33]. Ophthalmologic abnormalities associated with LS include optic atrophy, retinitis pigmentosa, strabismus, ptosis, nystagmus, and ophthalmoparesis. Pigmentary retinopathy was identified in patients with the *MT-ATP6* variant [34]. Movement disorders are commonly encountered in LS and include dystonia (resulting in involuntary muscle contractions, abnormal postures, and movements) and ataxia (resulting in gait instability and difficulties with fine motor tasks), the first being the most prevalent one [35]. Choreiform movements have been observed in Leigh syndrome, particularly in children with ATPase 6 mutations [36]. Peripheral nervous system manifestations include neuropathy and myopathy, although more commonly in Leigh-like syndromes. Myopathy in LS is characterized by muscle weakness, hypotonia, and exercise intolerance due to impaired oxidative phosphorylation within skeletal muscle fibers. Mutations in mitochondrial genes, such as *MT-ND5*, have been associated with myopathy [37].

### 3.2. Non-Neurological Manifestations

Non-neurological manifestations of LS may include dysmorphic features, although very rarely cardiac abnormalities (hypertrophic/dilated cardiomyopathy), and less frequently reported endocrine features (hypertrichosis and short height), gastrointestinal symptoms [8,38], diabetes mellitus, anemia, sleep disturbances, renal failure, hearing loss, and scoliosis. The majority of patients die before 3 years of age from sudden respiratory failure [39]. Cardiac manifestations, such as hypertrophic or dilated cardiomyopathy and conduction defects, have been reported in approximately 15% of individuals with LS [40]. Cardiomyopathy has been observed in up to 70% of individuals with an *MT-ND5* variant [41].

Gastrointestinal symptoms were even more prevalent in patients with COX-deficient Leigh syndrome caused by mutations in the *SURF1* gene [42]. Infants and children with Leigh syndrome often experience poor feeding, leading to failure to thrive. This can be due to central nervous system involvement, hypotonia, and poor suck-swallow coordination [42]. Gastroesophageal reflux, gastroparesis, and delayed gastric emptying are commonly observed and probably due to neuromuscular and mitochondrial dysfunction [43]. Hepatic manifestations, such as elevated liver transaminases, hepatomegaly, or liver failure, have been observed in about 10% of individuals with LS [44,45]. Renal abnormalities in Leigh syndrome are relatively uncommon, occurring in approximately 5% of affected individuals, according to reported cases. The primary renal manifestations include renal tubulopathy and glomerulocystic kidney disease [44,46,47]. Diabetes and short stature have also been documented [7]. While dysmorphic features are not commonly observed, they have been reported, especially in cases linked to PDH deficiency and *SURF1* mutations [48]. Hypertrichosis is a common feature in Leigh syndrome patients with *SURF1* mutations [49,50].

### 3.3. Leigh-Like Syndrome

Leigh-like syndrome has unusual neurological or radiological characteristics that share similarities with LS [51]. Individuals with this syndrome typically lack respiratory issues linked to brainstem involvement [52]. However, they may experience delayed onset of muscle weakness (myopathy) and minimal to no problems with eye movements [52]. Leigh-like syndrome may present itself with seizures (such as infantile spasms or hypsarrhythmia), alterations in behavior, decreased muscle tone (hypotonia), involuntary muscle contractions (dystonia), nerve-related problems (neuropathy), or abnormalities in the retina (retinopathy) [28]. Many experts believe Leigh syndrome and Leigh-like syndrome to be part of a disease continuum driven by shared or partially overlapping pathophysiological mechanisms and genetic mutations [50].

### 3.4. Presentation in Youth and Adults

A small subset of individuals diagnosed with either LS or Leigh-like syndrome manage to surpass the age of 10 years. Adolescent and adult-onset LS may arise in those with congenital LS who survive into adulthood, or when the syndrome first presents in late childhood or early adolescence [53]. An observational longitudinal cohort study enrolled seventy-two children with LS over 2.6 years, who completed the Newcastle Pediatric Mitochondrial Disease Scale (NPMDS), a scale used to quantify the disease burden and the rate of progression of LS [43]. The scale is composed of three sections, and each item has four responses: normal (0), mild (1), moderate (2), and severe (3) impairment. The total score from all three sections can be categorized into mild (0–14), moderate (15–25), and severe (>25). The median NPMDS score improved from 18 at baseline to 24 at follow-up. At the same time, the number of children needing gastrostomy or nasogastric tubes doubled, rising from 22.2% to 45.8%. The percentage of children experiencing epileptic seizures also increased, going from 29.2% to 37.5% [43]. During this time, twelve children died, and predictors of poor outcomes were *SURF1* gene variants and symmetrical hyperintensities on the caudate, globus pallidus, and putamen [43]. When LS manifests in adults, it is often associated with minimal neurological abnormalities [54] or with typical features consistent with LS or a mitochondrial disorder [53]. Patients with a primary coenzyme Q deficiency may exhibit symptoms consistent with adult LS [55]. Such individuals commonly display symptoms like encephalopathy, growth retardation, ataxia, and deafness [55]. Notably, neuropathological investigations conducted postmortem on a patient who had experienced a gradual progression of sensorimotor neuropathy, along with deafness, retinitis pigmentosa, and ataxia, showed characteristic LS lesions, undetectable on previous cerebral magnetic resonance imaging, despite their demise at age 37 [56].

## 4. Genetics

Leigh syndrome has been reported to be caused by defects in 16 mitochondrial genes and almost 100 nuclear genes [12]. MtDNA mutations seem to account for 21.5 to 47% of cases in many cohorts of genetically confirmed Leigh syndrome [47,57]. LS caused by mtDNA mutations can be maternally inherited or sporadic. Maternally inherited cases usually happen in a clinically unaffected mother. Inheritance of nuclear-encoded LS is typically autosomal recessive and rarely X-linked [12].

### 4.1. Complex I Deficiency

Complex I (CI) is the primary and largest complex, weighing approximately 1 MDa, whose main function involves the transfer of electrons from NADH to coenzyme Q10 while also transporting H+ ions. It consists of 44 subunits, including 14 core subunits crucial for its catalytic function, with seven subunits encoded by mtDNA and the remaining core subunits encoded by nDNA. Mutations in 24 CI subunit-encoding genes and several assembly factors have been linked to Leigh syndrome, highlighting the importance of CI in mitochondrial function. Notably, mutations in the nDNA-encoded *NDUFS4* gene, encoding subunit four of CI, induce ’mitochondrial complex I deficiency, nuclear type 1′ (*MC1DN1*), and Leigh syndrome in pediatric patients [58]. The lack of *NDUFS4* in various mouse tissues leads to reduced activity and stability of complex I. This instability causes a greater disconnect between electron influx from the NADH dehydrogenase module and the whole complex [58,59,60]. A dystonic later-onset form of *NDUFS4* was described with an onset of toes walking, dysarthric speech, nystagmus, and mental deterioration [61]. NADH ubiquinone oxidoreductase core subunit S8 (*NDUFS8*) is another crucial core component of the iron sulfide (FeS) fragment in mitochondrial complex I, playing a direct role in electron transfer and energy metabolism. Pathogenic variants of *NDUFS8* are associated with Leigh syndrome, as well as cancer and diabetes mellitus [61,62]. Several mtDNA mutations associated with Leigh syndrome are concentrated in MTND ‘hotspots’, particularly in *MTND1*, *MTND3*, and *MTND5* [63]. Isolated complex I deficiency is the most common oxidative phosphorylation enzyme defect, leading to a diverse clinical presentation that includes Leigh syndrome. The m.10191T>C mutation in the *MTND3* subunit is relatively frequent and can be either maternally inherited or a recurrent sporadic mutation. This mutation should be considered when evaluating individuals with complex I deficiency and Leigh syndrome features [64]. Defects of complex I are usually associated with visual disturbance, nystagmus, optic atrophy, and epilepsy [12].

### 4.2. Complex II–V Deficiency

Complex II subunits are all nuclear-encoded: four structural subunits (*SDHA*, *SDHB*, *SDHC*, and *SDHD*) and two known assembly factor genes (*SDHAF1* and *SDHAF2*). Complex II is distinct in that it serves as a component of both the respiratory chain and the Krebs cycle [65]. Mitochondrial diseases linked to isolated complex II deficiency are uncommon; they can result in Leigh syndrome or familial pheochromocytomas and paragangliomas [66]. Interestingly, in some cases of complex II deficiency, magnetic resonance spectroscopy of the brain can reveal a succinate peak [67].

Complex III defect involves proteins like *BCS1L*, *TTC19*, and in mouse models, *PARL*. Mutations in *TTC19* have been found to impair the function of Complex III, leading to a diverse clinical presentation that includes Leigh syndrome [68]. The clinical presentation of *TTC19* disease is variable and may consist of spinocerebellar ataxia and psychiatric manifestations, with the observation that hypertrophic olivary degeneration in the brain MRI may be of diagnostic value [69]. *PARL* deficiency, as shown in mouse models, leads to forms of Leigh-like syndrome [70]. A single variant in one subunit (UQCRQ) has been reported in more than 20 affected individuals of a consanguineous Israeli family [71].

Complex IV comprises three mtDNA subunits (COX I to III encoded by MT-CO1 to 3) and three nuclear-encoded subunits (*COX4I1*, *COX8A*, and *NDUFA4*). *NDUFA4* encodes a subunit of the respiratory chain Complex IV. *NDUFA4* dysfunction is recognized as the underlying cause of mitochondrial Complex IV deficiency nuclear type 21 (*MC4DN21*, OMIM 619065), a relatively mild presentation of Leigh syndrome [72]. *SURF1* is another involved protein in Complex IV deficiency and is one of the most common causes of LS. To date, over sixty distinct *SURF1* mutations have been identified as causes of *SURF1*-associated Leigh syndrome [73]. The majority of *SURF1*-associated Leigh syndrome cases follow a typical course, leading to early mortality before the age of ten. However, approximately 10% of cases exhibit an atypical progression with milder symptoms and a longer life expectancy [73]. Onset is usually in infancy with poor feeding and later on developmental regression. Ataxia, neuropathy ophthalmoplegia, and hypertrichosis are the most important manifestations [74]. In a murine model, *SURF1−/−* mice exhibited lower birth weights but eventually reached body weights similar to their siblings; however, they demonstrated a mild motor delay [75]. Other COX assembly factor defects associated with LS are deficiencies of *COX10*, *COX15*, *SCO2*, *PET100*, *PET117*, and *TACO1* [12].

Mitochondrial Complex V plays a crucial role in oxidative phosphorylation by facilitating ATP production. One of the first recognized genetic causes of LS was a recurrent single nucleotide mtDNA variant in the *MT-ATP6* gene, with a clinical spectrum varying according to the variable mutation load [76]. *ATP5PO*, which encodes the oligomycin sensitivity-conferring protein, is one of the proteins whose role has been recognized in Leigh syndrome presentation [77].

### 4.3. Other Mutations Associated with Leigh and Leigh-like Syndrome

LS is present in about one-third of cases of pyruvate dehydrogenase complex (PDHc) deficiency [78]. Five genetic defects of PDH have been linked to LS, such as *PDHA1*, *PDHB*, *PDHX*, *DLT*, and *DLD* mutations. The most frequently encountered is the *PDHA1* X-linked mutations [79]. These disorders do not always meet the clinical and pathological features for LS diagnosis. Disorders of vitamins and cofactor metabolism have also been associated with LS, such as biotinidase deficiency, which leads to biotin-dependent enzyme impairment [80]. Another treatable cause of LS is the *SLC19A13* mutation, which leads to Wernicke-like encephalopathy presentation when not fatal [81,82]. Disorders of mitochondrial DNA maintenance associated with LS include biallelic pathogenetic variants of *SUCLA2*, *SUCLG1*, *POLG*, and *RNASEH1*. The most relevant ones are *SUCL2* and *SUCLG1* mutations, which encode two subunits of succinyl-CoA ligase (a Krebs cycle enzyme that also helps with the maintenance of mtDNA). Patients with *SUCL2* variants usually have sensorineural hearing loss and dystonia, while *SUCLG1* patients have systemic involvement, especially in the liver and heart [83]. Many experts believe *POLG* mutations never meet the diagnostic criteria for LS, as they often present themselves with a clinically different spectrum, such as Alpers–Huttenlocher syndrome. Many other disorders of mitochondrial proteins have been linked to LS; among these, mutations in the *SERAC1* gene lead to 3-methylglutaconic aciduria, deafness, and encephalopathy, Leigh-like (MEGDEL) syndrome, which is a disorder of lipid remodeling [84].

### 4.4. Coenzyme Q Deficiency

Coenzyme Q (CoQ) deficiency can have different manifestations and can resemble LS [46]. For instance, a patient with coenzyme Q deficiency may exhibit congenital muscle weakness (hypotonia), seizures starting at age 3 months that are difficult to control and spread throughout the body, progressive muscle weakness, challenges in feeding, intermittent vomiting since age 7 months, the need for tube feeding, swelling due to low protein levels linked to poor feeding and kidney-related issues (nephrotic syndrome), ultimately resulting in death at 8 months [46]. In another case involving two sisters, primary coenzyme Q deficiency presented as an adult-onset syndrome characterized by brain dysfunction, slowed growth, lack of muscle coordination (ataxia), and hearing impairment [55]. Both individuals significantly improved with coenzyme Q replacement [55]. CoQ10 deficiency primarily affects the brain, muscles, and kidneys due to their high energy demand. Symptoms range from severe in infancy to mild in later life. It can cause ataxia, nephrotic syndrome, and hypertrophic cardiomyopathy. This entity should not be included in a Leigh-like syndrome per se.

## 5. Diagnosis

### 5.1. Laboratory Findings

Commonly, resting levels of lactate or pyruvate in the blood are elevated [8,27]. Elevated lactate levels (hyperlactatemia) may not be initially observed at the onset of symptoms but may develop as the disease progresses [85]. Moreover, in cases where skeletal muscles are affected, levels of creatine kinase are elevated [86]. Anemia has also been reported in isolated cases [85]. Patients with the Faroe variant typically display elevated levels of methylmalonic acid in their urine [11]. In cases of mitochondrial Leigh syndrome, there may be increased excretion of Krebs cycle intermediates in the urine. Lactate levels may be elevated in specific patients [87]. In patients with the Faroe variant, methylmalonic acid levels in the urine are typically high [11]. Patients with non-mitochondrial LS may display increased urinary excretion of 3-methylglutaconic acid or 3-methylglutaric acid [9,88]. In most cases, the levels of lactate, pyruvate, or the lactate/pyruvate ratio are elevated in the cerebrospinal fluid [8,89,90]. In a case involving a patient with Leigh syndrome resulting from a deficiency in coenzyme Q, there was a significant reduction in coenzyme Q levels [55].

### 5.2. Electrophysiological Assessments

Needle electromyography can detect irregular spontaneous activity [91]. Nerve-conduction studies have provided insights into potential polyneuropathy [91]. Auditory-evoked brainstem potentials may exhibit extended latencies even before clinical symptoms manifest [90,92,93]. Assessing visually evoked potentials may reveal elongation or reduction in amplitude of the P100 component [89], or in some cases, its complete absence [90]. Finally, an electroencephalogram may demonstrate irregular baseline activity and focal epileptic indicators, potentially leading to secondary generalization and hypsarrhythmia [86].

### 5.3. Neuroimaging

When clinical evaluations and laboratory tests trigger suspicion of LS, a cerebral MRI should be obtained. Distinctive observations in patients with LS typically include the presence of bilateral and symmetrical hyperintensities evident in T2-weighted images. These hyperintensities are primarily located in specific brain regions, notably the basal ganglia (especially the putamen) and various parts of the brainstem, such as the substantia nigra, nucleus ruber, and medulla oblongata [94,95]. Other findings include microcephaly, ventricular enlargement, intracranial pseudo-cysts, and white matter abnormalities [50]. Lesions in LS usually evolve over time; nonetheless, some case series reported partial or complete regression of lesions in follow-up MRI [39,95]. The reversibility of Leigh lesions underscores the importance of ongoing efforts to develop treatments that prevent brain damage [95]. Moreover, the absence of visible lesions does not rule out a diagnosis of Leigh syndrome, as patients may develop them later in the disease course [95]. It is also recommended to complement conventional MRI with proton magnetic resonance spectroscopy (MRS). The identification of a lactate peak in either brain parenchyma or cerebrospinal fluid (CSF) is considered a hallmark of mitochondrial disease.

Notably, there are correlations between MRI findings and the underlying genetic mutations in LS. For instance, in cases with complex I deficiency, brain MRIs often reveal bilateral symmetric brainstem lesions, at least one striatal anomaly, and an observable lactate peak in MRS [90]. Rarely, Leigh syndrome mimics tectal glioma, with symmetrical lesions in the midbrain and the pons, described as “giant panda” or “double panda sign” [96]. Furthermore, some patients with *POLG* and *SURF1* mutations have been reported to exhibit hypertrophic olivary degeneration, which may manifest clinically as tremors [15]. *POLG* mutations are associated with a variety of manifestations, in children in particular by Alpers–Huttenlocher disease or, later, by spinocerebellar ataxia and epilepsy. Therefore, these mutations are usually not included in the Leigh-like syndrome spectrum. Bindu and colleagues described the presence of bilateral hypertrophic olivary degeneration on MRI in a cohort of 10 children diagnosed with Leigh and Leigh-like syndrome [97]. Finally, the progression of LS over time is not always followed by a progression of lesions on MRI. Regression of lesions was demonstrated in five out of twelve patients [39]. Moreover, in another study, three patients had complete resolution of their lesions, demonstrating that LS lesions can be reversible and that the lack of lesions cannot rule out a diagnosis of LS [95].

### 5.4. Muscle Biopsies and Cultured Fibroblasts

Muscle tissue is frequently impacted in mitochondrial diseases due to its substantial energy requirements. Confirmation or exclusion of an OXPHOS disorder can be solely achieved through the analysis of a muscle biopsy [14]. An essential aspect of OXPHOS deficiency diagnosis involves the biochemical assessment of muscle biopsies extracted from either the quadriceps femoris muscle or the soleus muscle. To comprehensively assess the entire OXPHOS system, immediate processing of a freshly obtained biopsy is imperative. Notably, the necessity of a muscle biopsy in the diagnostic procedure is not universal. In cases where the clinical and biochemical phenotype strongly points to a specific mutation, it is advisable to commence a genetic analysis. Another valuable diagnostic tool is the examination of cultured fibroblasts derived from skin biopsies. This approach becomes pertinent when muscle tissue accessibility is limited or when validation and clarification of muscle biopsy results are required. However, it’s worth noting that OXPHOS disorders in muscle tissue may exhibit less marked symptoms or even be absent in cultured fibroblasts.

### 5.5. Genetic Diagnosis

Mitochondrial disorders occur with various clinical and biochemical features, implicating hundreds of genes present in both mtDNA and nDNA. When clinical features lack specificity or a multitude of candidate genes exist, a broader approach is necessary. A primary step in genetic diagnosis involves full sequence analysis of mtDNA, often extracted from affected tissue, primarily muscle. This approach is particularly useful for identifying mutations with high heteroplasmy levels. In cases of mtDNA depletion without detectable mutations, consideration of *POLG* gene mutations, encoding polymerase gamma essential for mtDNA replication and repair, becomes crucial. If mtDNA mutations are ruled out, the focus shifts to sequencing candidate nuclear genes based on the patient’s clinical and biochemical features. The conventional strategy involves sequencing the most frequently mutated genes among the selected candidates. However, nowadays next-generation sequencing (NGS) technologies enable the sequencing of multiple candidate genes or even entire exomes.

### 5.6. Genetic Counseling

Genetic counseling is crucial for parents with children affected by LS who want to elucidate the underlying genetic factors. Categorizing mitochondrial disorders based on the identified mutations—whether in nuclear-encoded or mtDNA-encoded genes—provides a framework for risk assessment and future family planning.

Table 1 provides information on the known mtDNA and nDNA genes responsible for LS.

### 5.7. Differential Diagnosis

Several disorders share clinical features with LS, necessitating a meticulous medical history and targeted laboratory investigations. Key differential diagnoses include neonatal asphyxia, Wernicke encephalopathy, various metabolic disorders, and toxic-induced basal ganglia lesions.

## 6. Established Therapy

To date, no definitive treatment has been identified for LS. Current therapies include supplements (coenzyme Q10 and its derivatives), vitamins, pyruvate, dichloroacetate, and a ketogenic diet. Table 2 provides information on the current drugs and therapies used for LS and preclinical in vivo studies. However, recent systematic reviews have highlighted additional therapeutic interventions under investigation, including experimental treatments and clinical trials [99].

### 6.1. Coenzyme Q10 and Vatiquinone

Coenzyme Q10, also called ubiquinone, plays a vital role in shuttling electrons between complex II and III. In a specific case study, a patient with LS and an m.10197 G>A mutation experienced notable improvement after receiving CoQ10 supplementation for three months [100]. Furthermore, an MRI scan conducted during a one-year follow-up showed the complete disappearance of the previously observed lesion. It is worth noting, however, that CoQ10 supplementation does not consistently produce positive results in cases where there is a CoQ10 deficiency [46]. Six studies evaluated CoQ10 as a standalone treatment, with four reporting clinical improvement in patients harboring mutations in m.10197G>A, *COQ2*, and *COQ4* [101]. However, it was observed that patients in advanced stages of the disease showed clinical deterioration despite CoQ10 initiation, underscoring the importance of early intervention [101]. In some cases, renal function improvements were noted following CoQ10 supplementation [102]. Conversely, Scalais et al. reported a case of a child who developed severe proteinuria despite receiving CoQ10 therapy from infancy [103].

Some patients with LS have responded positively to alternative treatments such as EPI-743 (Vatiquinone) and idebenone [104]. EPI-743 is a para-benzoquinone that repletes intracellular glutathione more potently than coenzyme Q10 or idebenone. It has been tested in open-label clinical trials for mitochondrial diseases, including LS. Enns et al. found that 11 out of 12 participants showed clinical and radiological improvements [105]. Additionally, Martinelli et al. observed significant improvements in 10 children with genetically confirmed LS treated with EPI-743 [106]. While EPI-743 appears to have broad efficacy across genetic variations, and other drugs are currently undergoing clinical trials, not all outcomes have been satisfactory [46].

### 6.2. Sodium Dichloroacetate and Sodium Pyruvate

Another compound, dichloroacetate (DCA), enhances pyruvate dehydrogenase activity by reducing lactate buildup. DCA treatment has shown effectiveness in patients with LS with the T8993C mutation in *ATPase 6*, a subunit of complex V [107], as well as PDHc and CI deficiency [108]. However, studies have reported mixed clinical outcomes with DCA treatment. While it has shown potential benefits, DCA is also associated with the risk of worsening peripheral neuropathy, even when thiamine is administered prophylactically to mitigate side effects [109]. For instance, Fujii et al. documented successful improvement in midbrain hyperintensities in a patient with the *MT-ATP6* m.8993T>C mutation following DCA and thiamine therapy [107]. In contrast, Koga et al. described a case of a child with a *PDHE1* mutation who experienced clinical deterioration with DCA but subsequently responded positively to pyruvate therapy [110].

It is also important to mention pyruvate, which plays a crucial role in remedying the malfunction of glyceraldehyde-3-phosphate dehydrogenase (GAPDH) by supplying NAD, thus restoring the NADH/NAD+ ratio that becomes disrupted in disorders marked by OXPHOS deficiency [111]. In a specific case study, the oral administration of sodium pyruvate over one year significantly enhanced exercise tolerance, ventricular ejection fraction, and the normalization of an abnormal echocardiographic presentation [112]. Most studies focused on biochemical pyruvate dehydrogenase deficiencies, which were not confirmed radiologically or genetically as Leigh syndrome (LS). Only two papers met all inclusion and exclusion criteria. The highest level of evidence came from a case series by Fujii et al., which included two genetically confirmed patients with LS with the variants m.8993T>G and m.9176T>C. Both patients, who were bedbound, showed improvements in the Newcastle Pediatric Mitochondrial Disease Scale (NPMDS) following pyruvate treatment [113].

### 6.3. Sonlicromanol

Sonlicromanol (KH176) is a promising redox-modulating agent under investigation for mitochondrial diseases, including Leigh syndrome (LS). A Phase 1 study (NCT02544217) evaluating KH176 in patients with various mitochondrial disorders, including LS, demonstrated that the agent was generally well tolerated. However, at higher doses, it was associated with QTc prolongation and T-wave morphological changes, necessitating careful dose optimization and monitoring [114]. Building on these findings, randomized, multi-center Phase 2 trials are currently recruiting participants with a range of mitochondrial disorders, such as MELAS (mitochondrial encephalomyopathy, lactic acidosis, and stroke-like episodes), MIDD (maternally inherited diabetes and deafness), LS, mitochondrial myopathies, and mitochondrial encephalopathies. These studies aim to evaluate further the safety and efficacy of KH176 across different mitochondrial disease phenotypes. Notably, a safety and efficacy trial focusing on m.3243A>G-associated mitochondrial disease has already been completed, providing valuable insights into the therapeutic potential of KH176 in this specific genetic context [115].

### 6.4. Vitamin Supplementation

Vitamin supplementation is another approach to ameliorate the neurological symptoms of LS. High doses of riboflavin enhance muscle strength and alleviate lactic acidosis in the context of complex I deficiency due to mutation in *ACAD9* mutation, which is a crucial factor for the operation of the OXPHOS system and ATP production [115,116]. Nonetheless, *ACAD9* mutation seems to show a different pathology from the one observed in LS; *ACAD9* mutations are associated with a non-Leigh complex encephalopathy and often cardiomyopathy in most cases. Riboflavin was utilized in 18 studies, though it was predominantly administered as part of a “mitochondrial cocktail” rather than as a standalone treatment. Only three studies specifically evaluated riboflavin monotherapy. Two studies demonstrated clinical improvement in patients with ACAD9 mutations [115,117]. Notably, Gerards et al. reported the restoration of complex I activity in a family with a homozygous *ACAD9* (c.1594C>T) mutation following riboflavin monotherapy [115].

Moreover, early supplementation with both thiamine and biotin in patients with LS caused by a mutation in *SLC19A3*, a gene essential to thiamine transporter-2 deficiency, has been found to have an immediate therapeutic effect [118,119]. The *SLC19A3* gene encodes a thiamine transporter, and mutations in this gene are associated with Biotin-Responsive Basal Ganglia Disease (BBGD). A review of 22 and 57 studies revealed that biotin and thiamine supplementation are commonly used as part of a “mitochondrial cocktail” for treating mitochondrial disorders. However, only two case series have directly compared biotin or thiamine monotherapy versus combination therapy. Debs et al. reported clinical and radiological improvement in two siblings with *SLC19A3* missense mutations, both with biotin monotherapy and combination therapy [120]. In contrast, Tabarki et al. found no significant difference in the Burke–Fahn–Marsden Dystonia Rating Scale (BFMDRS) scores between thiamine monotherapy and combination therapy in an open-label prospective study involving 20 children with the *SLC19A3* c.1264A>G (p.Thr422Ala) mutation. However, combination therapy was associated with a significantly faster recovery from acute crises (2 days vs. 3 days) compared to monotherapy [121].

### 6.5. N-Acetylcysteine and Carnitine

N-acetylcysteine (NAC) is a compound that serves as a precursor to glutathione, a powerful antioxidant that helps neutralize toxic sulfides [122]. In one study, Viscomi et al. reported positive outcomes in five children with homozygous 505 + 1G>T splice-site mutations in the *ETHE1* gene, responsible for a syndrome that shows a different pathology but shares many similarities with LS. These children experienced a reduced seizure frequency, neurological improvements, and decreased symptoms such as acrocyanosis (bluish discoloration of extremities) and petechiae (small red or purple spots on the skin). Additionally, their episodes of diarrhea improved, which was likely enhanced by the combined use of Metronidazole, a bactericidal and prokinetic agent [123]. Another case, described by Shayota et al., documented developmental improvements in a patient with an *ECHS1* mutation following NAC treatment. However, the patient was also on a valine-restricted diet, making it difficult to determine the exact contribution of NAC to the observed progress [124]. While these findings are encouraging, further studies are needed to clarify NAC’s role in managing mitochondrial disorders and to determine its effectiveness as a standalone treatment.

Carnitine plays a crucial role in fatty acid oxidation. It is commonly included in mitochondrial cocktail therapies with combinations of nutraceuticals, cofactors, and antioxidants to support mitochondrial function and bypass defects in the electron transport chain [125]. However, its effectiveness remains uncertain, as many case series and reports have documented its use with little to no noticeable benefit [101]. One notable case, described by Toth et al., involved a patient with the m.8993T>C mutation who experienced sudden loss of mobility following an upper respiratory tract infection. The patient’s condition improved after receiving carnitine supplementation, but it is unclear whether this was due to the treatment itself or simply the natural resolution of the infection. The authors noted that the patient had low plasma and muscle carnitine levels before treatment. Later, at age 13, the patient experienced worsening ataxia and muscle weakness, which improved after the carnitine dose was adjusted based on body weight [126]. While carnitine remains a widely used supplement in mitochondrial disease management, further studies are needed to determine its actual therapeutic value, particularly regarding disease progression and symptomatic relief.

### 6.6. Ketogenic Diet

KD seems to extend longevity and ameliorate mental problems [127]. Moreover, it appears to reverse the oculomotor palsy of LS caused by the pathogenic *NDUFV1* variant. Ketogenic diets are believed to enhance fatty acid β-oxidation, providing an alternative energy pathway when oxidative phosphorylation is impaired [128]. These diets have shown effectiveness in controlling epilepsy, improving eye movements, and supporting mental development in patients with mutations in genes such as *ECHS1*, *POLG*, *TMEM126B*, and *PDHA 1*, which causes pyruvate dehydrogenase deficiency [101]. Additionally, protein and valine-restricted diets have been reported to benefit patients with mutations affecting valine degradation pathways, such as those in *HIBCH* and *ECHS1* [128]. These dietary interventions prevent the buildup of toxic metabolites like methacrylyl-CoA and acryloyl-CoA [124]. However, ketogenic diets can also pose risks, such as inducing metabolic acidosis and potentially worsening clinical symptoms in some patients with LS [129].

## 7. Promising Therapy

### 7.1. Spindle Nuclear Transfer

An innovative technology called spindle nuclear transfer (SNT) for females with LS prevents vertical transmission of mutated mtDNA. SNT consists of transferring the nucleus to another egg from a healthy donor with normal mitochondria but stripped of its nucleus. This remarkable technique holds great promise in mitochondrial disorders, although ethical concerns have been raised [130], and long-term follow-up of the child’s longitudinal development remains to be seen [131]. The spindle transfer is a preventative approach that has been prohibited in the US. In contrast, the UK and Australia have adopted the pronuclear transfer, but so far, no positive publication about the clinical efficacy of this approach has been released.

### 7.2. Gene Therapy

Gene therapy holds great potential for treating orphan diseases, particularly with the advent of precision medicine. Administering adenovirus-associated virus (AAV) has been shown to alleviate respiratory abnormalities and lead to mild clinical improvements in *NDUFS4* KO mice [132]. Adeno-associated virus (AAV) recombinant vectors have emerged as a promising gene therapy approach for LS. AAV vectors are particularly advantageous due to their ability to transduce post-mitotic cells, including neurons, with long-term gene expression and minimal immunogenicity. In the context of Leigh syndrome, AAV-mediated gene therapy aims to deliver functional copies of nuclear-encoded mitochondrial genes or introduce therapeutic molecules that enhance mitochondrial function. Studies exploring AAV-based interventions have shown potential in preclinical models, particularly in targeting defects associated with complex I deficiencies, the most common cause of Leigh syndrome. However, challenges remain, including optimizing tissue-specific targeting, overcoming the blood-brain barrier, and ensuring sustained expression without adverse immune responses. As research advances, AAV vectors hold promise for the development of targeted therapies and, as of now, represent the only possibility for a cure as they convey the missing gene to the brain [133].

Interestingly, findings from a recent study indicate that new approaches involving mitochondrial transfer hold potential as a therapeutic strategy for treating mitochondrial diseases, including Leigh syndrome [134].

### 7.3. Disease Models of Leigh Syndrome

Various disease models of LS have been developed, leading to essential findings into LS pathophysiology, with the most prominent model of LS being the *NDUFS4* knockout mouse model [135]. *NDUFS4* is a nuclear gene encoding the NADH-dehydrogenase subunit S4 of the OXPHOS complex I; its mutation leads to mitochondrial dysfunction. Mitochondrial dysfunction in neurons can lead to lipid droplets (LD) buildup in glial cells and may occur before LS symptoms appear. Mouse models of *NDUFS4* mutations developed bilateral spongiform lesions, and systemic inflammation which seems to result from LD accumulation in glial cells [60]. In both mouse and Drosophila models, LD accumulation was associated with increased reactive oxygen species (ROS) levels, and both effects were alleviated by antioxidants. This indicates that oxidative stress can drive lipid droplet accumulation during mitochondrial dysfunction. Consequently, ROS-induced lipid peroxidation and subsequent neuroinflammation may contribute to the underlying disease mechanism [58]. These mouse models show how mTOR inhibition and antioxidants could be a promising approach to mitigate the severe phenotype in patients with Leigh syndrome. Treating *NDUFS4* mice with AD4 helped reduce LD levels, slightly delaying the onset of neurodegenerative signs and improving motor function [60]. Repletion of nicotinamide adenine dinucleotide (NAD+) granted extended lifespan in murine models of *NDUFS4* [136]. Compounds such as rapamycin and doxycycline improved and doubled the lifespan of the *NDUFS4* knockout mouse model [137,138,139]. Mouse models by the Mootha lab have also demonstrated that one of the most effective interventions in *NDUFS4* models is prolonged, continuous exposure to mild hypoxia, resulting in a significant lifespan extension and pathological and radiological improvement [140,141,142,143].

Yeast models have also been developed, among these, the S. cerevisiae model of deficiency by editing the *SURF1* homolog Shy1 helped confirm the importance of this assembly factor of the complex IV [144]. Zebrafish *SURF1* models provided evidence for cysteamine bitartrate and N-Acetylcysteine in ameliorating ROS in *SURF1*-associated LS [145]. Non-mammalian animal models have also been used in LS. Burman et al. generated a Drosophila model carrying a deletion in the *MT-ND2* gene [146]. This knockout model revealed complex I reduced activity with features of mitochondrial disease. This model was used to test rapamycin as a potential treatment, with evidence of fat storage defect rescue [147]. In *Drosophila*, mutations in the ND23 subunit of mitochondrial complex I—analogous to the mammalian *NDUFS8*—mimic key features of LS. Mutations in the mitochondrial complex I subunit ND23 enhance susceptibility to isoflurane-induced toxicity and oxidative stress in *Drosophila*. Asymptomatic flies carrying *ND23* and *MT-ND2* mutations become increasingly vulnerable to isoflurane and halothane toxicity due to aging and genetic background [148,149]. This model helped clinicians understand the danger related to anesthetic usage in patients with LS. Also, patient fibroblast models carrying mtDNA mutations in *MT-ND1*, three and five were helpful in showing possible therapeutic compounds such as hispidin, a natural fungal compound, which helped inhibit the pro-oxidative pathway mediated by p66Shc protein [150]. Another LS disease model came after discovering a method for reprogramming fibroblast cultures into induced pluripotent stem cells (iPSCs) [151]. An iPSC model for *MT-ATP6* mutation showed some benefit in helping mitochondrial membrane potential restoration with the PDE5 inhibitor avanafil [152]. An iPSCs model of *NDUFS4* helped provide evidence for nicotinamide riboside (NR) as a possible treatment strategy for LS [153]. NR, a vitamin B3 analogue and precursor of nicotinamide adenine dinucleotide (NAD+), supports beneficial effects in LS through a mechanism dependent on acetylation. Targeted metabolomic analysis of heart and brain samples from LS mice, both under basal conditions and after NR supplementation, revealed disruptions in NAD+-dependent metabolic enzymes. These disruptions were restored following NR treatment. As NAD+ serves as a cosubstrate for Sirtuin deacetylases, its depletion leads to reduced Sirtuin 1 (SIRT1) activity, resulting in increased protein acetylation [154]. Possible therapies to ameliorate SIRT1 activity are discussed in the following section.

### 7.4. Sirtuins

Sirtuins (SIRTs) are a class of histone deacetylases that rely on nicotinamide adenine dinucleotide (NAD+) as a cofactor. They play crucial roles in regulating vital signaling pathways in both prokaryotes and eukaryotes, contributing to various biological processes. Currently, seven mammalian homologs of yeast Sir2 are known as SIRT1 to SIRT7. These proteins are involved in various essential cellular processes, including inflammation, metabolism, oxidative stress, and apoptosis. Consequently, they are considered promising therapeutic targets for various pathologies, including cancer, cardiovascular disease, respiratory issues, and other medical conditions. SIRT1, for example, regulates mitochondrial energy metabolism and is known to boost mitochondrial biogenesis by activating PGC-1α [155,156]. One emerging strategy in LS involves activating SIRT1, a key regulator of cellular metabolism and mitochondrial function. SIRT1 activation has been explored using NAD+ precursors and poly(ADP-ribose) polymerase (PARP) inhibitors, which enhance mitochondrial biogenesis and function. Enhancing SIRT1 activity may counteract mitochondrial dysfunction in LS by promoting energy homeostasis, reducing oxidative stress, and increasing cellular resilience [157]. NAD+ is a crucial coenzyme in mitochondrial metabolism and a direct modulator of SIRT1 activity. NAD+ precursors, such as nicotinamide riboside and nicotinamide mononucleotide, have been shown to boost NAD+ levels, thereby enhancing SIRT1 function. Studies in mitochondrial disease models indicate that NAD+ supplementation improves mitochondrial respiration and reduces neurodegeneration [138]. Ongoing clinical trials are assessing the benefits of NAD+ augmentation in mitochondrial diseases, including Leigh syndrome, with promising preliminary results. Another strategy to preserve NAD+ levels involves the inhibition of PARP. PARP enzymes play a critical role in DNA repair but excessively consume NAD+, leading to energy depletion in mitochondrial diseases. PARP inhibitors (PARPis) such as olaparib and veliparib have been investigated for their ability to prevent NAD+ depletion and enhance mitochondrial function. PARP inhibition has been reported to improve cellular energy balance and mitochondrial dynamics in neurodegenerative condition models [157]. By preserving NAD+ levels, PARP inhibitors may sustain SIRT1 activity and improve mitochondrial resilience in patients with LS. Therapies targeting SIRT1 activation, including NAD+ precursors and PARP inhibitors, offer a promising avenue for mitigating mitochondrial dysfunction in Leigh syndrome. By enhancing mitochondrial biogenesis, reducing oxidative stress, and preserving cellular energy balance, these approaches hold potential for improving patient outcomes.

### 7.5. Rapamycin

Rapamycin, known as sirolimus, is commonly administered to prevent immunological rejection after kidney transplantation. Rapamycin, a macrolide antibiotic and potent inhibitor of the mechanistic target of rapamycin (mTOR), has shown therapeutic potential in preclinical models of mitochondrial dysfunction [158]. One of the most significant mechanisms through which rapamycin exerts neuroprotective effects is by enhancing autophagy and mitophagy. In mitochondrial diseases, dysfunctional mitochondria accumulate, contributing to oxidative damage and cellular dysfunction. Rapamycin-induced autophagy facilitates the degradation and recycling of damaged mitochondria, thereby improving cellular homeostasis. Increased mitophagy helps to remove defective mitochondria, reducing the burden of oxidative stress and improving mitochondrial network function [158]. Rapamycin mitigates oxidative stress by upregulating antioxidant defense mechanisms, such as increased expression of superoxide dismutase (SOD) and nuclear factor erythroid 2-related factor 2 (NRF2). Additionally, rapamycin modulates inflammatory pathways by reducing the activation of pro-inflammatory cytokines and microglial activation, thereby limiting neuroinflammation, which is a key contributor to neurodegeneration in complex I deficiency [136]. Rapamycin has also been shown to influence the integrated stress response (ISR), a cellular pathway activated in response to mitochondrial dysfunction. By modulating ISR signaling, rapamycin promotes cell survival and adaptation to mitochondrial stress, improving neuronal resilience in complex I-defective models [136]. In a mouse model designed to simulate LS, rapamycin showed promising results by improving neurological symptoms and extending the lifespan to approximately 110 days, twice as long as the untreated control group, whose lifespan was around 50 days [159]. Rapamycin also emulates the effects of caloric restriction (CR), a practice known to extend lifespan and reduce the risk of age-related health issues in yeast [160]. However, it’s essential to acknowledge that there are prevalent side effects associated with rapamycin usage, including hyperlipidemia, immunosuppression, and impaired wound healing. These side effects can present challenges, particularly when considering their long-term use [161].

### 7.6. From Preclinical Model to New Interventions

Preclinical models have helped gain insight into new possible therapeutic options. Treatment of LS usually involves vitamins, supplementation, and a ketogenic diet [135]. Experimental models provided preclinical evidence for human trials for the mTOR inhibitors, such as the nab-sirolimus trial (NCT03747328), recently withdrawn, and sirolimus (NCT06843811) currently enrolling. Another EPI-743 trial was recently completed (NCT02352896), and results are waiting to be posted. The European Medical Agency (EMA) issued an orphan drug designation for cannabidiol (EU/3/23/2800) and sildenafil (EU/3/23/2831), both showing some efficacy in mouse models [152,162]. Gene replacement therapy using the AAV9/hSURF1 vector in patients with LS with *SURF1* mutations has obtained an orphan drug designation by EMA (EU/3/21/2531) after proving successful in *SURF1* knockout mice [98].

## 8. Prognosis and Conclusions

Despite notable progress in medical treatments, the outlook for individuals with Leigh syndrome remains a complex and challenging one. Regrettably, a significant portion of cases result in fatality, often occurring before the age of five. Nevertheless, by implementing vigilant monitoring of patients who exhibit respiratory problems and employing a range of diagnostic assessments to assess brainstem function, including magnetic resonance imaging, auditory-evoked brainstem potentials, somatosensory-evoked potentials, blink reflex, or polysomnography, there is the potential to prevent sudden and early fatalities in individuals with early-onset Leigh syndrome. However, most of the interventions are palliative and not radical treatments. Among current treatments, AAV vectors hold promise for the development of targeted therapies and, as of now, represent the only possibility for a cure as they convey the missing gene to the brain. Further clinical studies are needed to evaluate the safety and efficacy of gene therapy.

## Figures and Tables

**Table 1:** Summary of genes involved in the Leigh Syndrome (LS).

Inheritance	
Autosomal Recessive/ (Maternal/Sporadic)	Genetic Mutations
Complex I	*NDUFS1*, *NDUFS2*, *NDUFS3*, *NDUFS4*, *NDUFS7*, *NDUFS8*, *NDUFV1*, *NDUFV2*, *NDUFA2*, *NDUFA9*, *NDUFA10*, *NDUFA12*, *NDUFA13*, *NDUFAF2*, *NDUFAF4*, *NDUFAF5*, *NDUFAF6*, *FOXRED1*, *NUBPL*, *NDUFAF8*, *TIMMDC1*, *NDUFB8*, *NDUFC2* *(MT-ND1*, *MT-ND2*, *MT-ND3*, *MT-ND4*, *MT-ND5,* *MT-ND6)*
Complex II	*SDHA*, *SDHAF1* *
Complex III	*UQCRQ*, *TTC19*, *BCS1L*
Complex IV	*SURF1*, *NDUFA4*, *COX4I1*, *COX8A*, *COX10*, *COX15*, *SCO2*, *LRPPRC*, *TACO1*, *PET100*, *PET117* *(MT-CO1*, *MT-CO2*, *MT-CO3)*
Complex V	*ATP5MD (MT-ATP6)*
mitochondrial DNA maintenance	*POLG* *, *SUCLA2*, *SUCLG1*, *FBXL4*, *SLC25A4*, *SSBP1*, *RNASEH1*, *GTPBP3*
mitochondrial gene expression	*TRMU*, *GTPBP3*, *MTFMT*, *EARS2*, *FARS2*, *IARS2*, *NARS2*, *PTCD3*, *MRPS34*, *GFM1*, *GFM2*, *TSFM*, *MTRFR*, *PNPT1*, *C12ORF65*, *TARS2* *(MT-TI*, *MT-TK*, *MT-TL1*, *MT-TL2*, *MT-TV*, *MT-TW)*
mitochondrial cofactor	*PDSS2*, *COQ9*, *LIAS*, *LIPT1*, *MECR*
mitochondrial membrane	*SERAC1*, *MFF*, *SLC25A46*, *CLPB*
mitochondrial toxicity	*HIBCH*, *ECHS1*, *ETHE1* *, *SQOR*, *SLC39A8*, *NAXE*
mitochondrial (other)	*LONP1*, *VPS13D*, *OPA1*
pyruvate dehydrogenase complex	*PDHB*, *DLAT*, *DLD*, *PDHX*
B vitamin transport and metabolism	*SLC25A19*, *TPK1*, *BTD*, *SLC19A3*
miscellaneous	*HPDL*, *ADAR*, *NUP62*, *RANBP2*, *MORC2*
Autosomal dominant	*DNM1L*
X-linked	*PDHA1* *, *NDUFA1*, *AIFM1* *

* These mutations include syndromes that may resemble Leigh syndrome but show a different pathology. Ethylmalonic encephalopathy (due to mutations of ETHE1) has a distinct, complex neuropathology evidenced by both autoptic and MRI examination. POLG mutations are associated with a variety of manifestations, in children in particular by Alpers–Huttenlocher disease or, later, by Spinocerebellar ataxia and epilepsy. Therefore, these mutations are not usually included in the Leigh-like syndrome spectrum. Defects in PDHA can also appear as Leigh syndrome at MRI, but they are typically associated with other lesions, such as micropore accumulation, especially in the frontal region. Table adapted from [98].

**Table 2:** Proposed therapies and target mutation/pathways.

Treatment	Specific Mutations or Deficiencies Reported
Coenzyme Q10	*ND3*-m.10197 G>A, Succinate: cytochrome c oxidoreductase deficiency, m.9185 T>C, m.10191 T>C, *PDSS2*/CoQ10 deficiency
EPI-743	ND1-G3697A, *SUCLA2*, *ETHE1* *, ND5-G13513A, *EARS2*, *SURF1*, ND1-G3697A, ND6-T14487C
Idebenone	Unknown
KH176	Mitochondrial m.3243A>G Spectrum Disorders
Riboflavin	*ACAD9* *- c.1240C>T
Thiamine	*SLC19A3*/Thiamine transporter-2 deficiency
Thiamine + Biotin + CoenzymeQ10 + Vitamin E + Vitamin C + Carnitine	*SLC19A3*/Thiamine transporter-2 deficiency
Sodium Pyruvate	Unknown, m.8993 T>G, m.9176 T>C
Sodium Dichloroacetate	ATP6-m.8993 T>C, ATP6-m.8993 T>G
N-acetylcysteine	*ETHE1* *
Carnitine	m.8993T>C mutation
Ketogenic diet	PDHc deficiency, *NDUFV1*
Plasmapheresis + IVIG	*ATP6*-m.9176 T>C
Preclinical Studies	
	Targeted mechanism
Rapamycin	Unknown, mTOR pathway
Hypoxia	Oxygen restriction
AVV gene therapy	Insertion of a target gene
Olaparib, veliparib	PARP inhibitors (PARPis)
Nicotinamide riboside and nicotinamide mononucleotide	(NAD+ precursors) enhancing SIRT1 function
AD4	Lipid metabolism
Hispidin	MTND
Avanafil	*MT-APT6* mutation, membrane potential

* These mutations include syndromes that may resemble Leigh syndrome but show a different pathology: *ACAD9* mutations are associated with a non-Leigh complex encephalopathy and often cardiomyopathy. Adapted from [24].

## Data Availability

Data are contained within the article.

## References

[1] Schubert Baldo M., Vilarinho L. (2020). Molecular basis of Leigh syndrome: A current look. Orphanet J. Rare Dis..

[2] Balasubramaniam S., Lewis B., Mock D.M., Said H.M., Tarailo-Graovac M., Mattman A., van Karnebeek C.D., Thorburn D.R., Rodenburg R.J., Christodoulou J. (2017). Leigh-Like Syndrome Due to Homoplasmic m.8993T > G Variant with Hypocitrullinemia and Unusual Biochemical Features Suggestive of Multiple Carboxylase Deficiency (MCD). JIMD Rep..

[3] Ogier H., Lombes A., Scholte H.R., Poll-The B.T., Fardeau M., Alcardi J., Vignes B., Niaudet P., Saudubray J.M. (1988). de Toni-Fanconi-Debré syndrome with Leigh syndrome revealing severe muscle cytochrome c oxidase deficiency. J. Pediatr..

[4] Lake N.J., Compton A.G., Rahman S., Thorburn D.R. (2016). Leigh syndrome: One disorder, more than 75 monogenic causes. Ann. Neurol..

[5] Kucharczyk R., Rak M., di Rago J.P. (2009). Biochemical consequences in yeast of the human mitochondrial DNA 8993T > C mutation in the ATPase6 gene found in NARP/MILS patients. Biochim. Biophys. Acta.

[6] Baertling F., Rodenburg R.J., Schaper J., Smeitink J.A., Koopman W.J., Mayatepek E., Morava E., Distelmaier F. (2014). A guide to diagnosis and treatment of Leigh syndrome. J. Neurol. Neurosurg. Psychiatry.

[7] Finsterer J. (2008). Leigh and Leigh-like syndrome in children and adults. Pediatr. Neurol..

[8] Chol M., Lebon S., Bénit P., Chretien D., de Lonlay P., Goldenberg A., Odent S., Hertz-Pannier L., Vincent-Delorme C., Cormier-Daire V. (2003). The mitochondrial DNA G13513A MELAS mutation in the NADH dehydrogenase 5 gene is a frequent cause of Leigh-like syndrome with isolated complex I deficiency. J. Med. Genet..

[9] Di Rocco M., Caruso U., Moroni I., Lupino S., Lamantea E., Fantasia A.R., Borrone C., Gibson K.M. (1999). 3-Methylglutaconic aciduria and hypermethioninaemia in a child with clinical and neuroradiological findings of Leigh disease. J. Inherit. Metab. Dis..

[10] Cooper M.P., Qu L., Rohas L.M., Lin J., Yang W., Erdjument-Bromage H., Tempst P., Spiegelman B.M. (2006). Defects in energy homeostasis in Leigh syndrome French Canadian variant through PGC-1alpha/LRP130 complex. Genes Dev..

[11] Rahman S., Blok R.B., Dahl H.H., Danks D.M., Kirby D.M., Chow C.W., Christodoulou J., Thorburn D.R. (1996). Leigh syndrome: Clinical features and biochemical and DNA abnormalities. Ann. Neurol..

[12] Rahman S., Horvath R., Hirano M., Chinnery P.F. (2023). Chapter 4—Leigh syndrome. Handbook of Clinical Neurology.

[13] Ostergaard E., Hansen F.J., Sorensen N., Duno M., Vissing J., Larsen P.L., Faeroe O., Thorgrimsson S., Wibrand F., Christensen E. (2007). Mitochondrial encephalomyopathy with elevated methylmalonic acid is caused by SUCLA2 mutations. Brain.

[14] Morin C., Mitchell G., Larochelle J., Lambert M., Ogier H., Robinson B.H., De Braekeleer M. (1993). Clinical, metabolic, and genetic aspects of cytochrome C oxidase deficiency in Saguenay-Lac-Saint-Jean. Am. J. Hum. Genet..

[15] Merante F., Petrova-Benedict R., MacKay N., Mitchell G., Lambert M., Morin C., De Braekeleer M., Laframboise R., Gagné R., Robinson B.H. (1993). A biochemically distinct form of cytochrome oxidase (COX) deficiency in the Saguenay-Lac-Saint-Jean region of Quebec. Am. J. Hum. Genet..

[16] Oláhová M., Hardy S.A., Hall J., Yarham J.W., Haack T.B., Wilson W.C., Alston C.L., He L., Aznauryan E., Brown R.M. (2015). LRPPRC mutations cause early-onset multisystem mitochondrial disease outside of the French-Canadian population. Brain.

[17] Hong C.-M., Na J.-H., Park S., Lee Y.-M. (2020). Clinical Characteristics of Early-Onset and Late-Onset Leigh Syndrome. Front. Neurol..

[18] Debray F.G., Morin C., Janvier A., Villeneuve J., Maranda B., Laframboise R., Lacroix J., Decarie J.C., Robitaille Y., Lambert M. (2011). LRPPRC mutations cause a phenotypically distinct form of Leigh syndrome with cytochrome c oxidase deficiency. J. Med. Genet..

[19] Leigh D. (1951). Subacute necrotizing encephalomyelopathy in an infant. J. Neurol. Neurosurg. Psychiatry.

[20] Arii J., Tanabe Y. (2000). Leigh syndrome: Serial MR imaging and clinical follow-up. Am. J. Neuroradiol..

[21] Piao Y.S., Tang G.C., Yang H., Lu D.H. (2006). Clinico-neuropathological study of a Chinese case of familial adult Leigh syndrome. Neuropathology.

[22] Koenig M.K. (2008). Presentation and diagnosis of mitochondrial disorders in children. Pediatr. Neurol..

[23] Lee J.S., Yoo T., Lee M., Lee Y., Jeon E., Kim S.Y., Lim B.C., Kim K.J., Choi M., Chae J.H. (2020). Genetic heterogeneity in Leigh syndrome: Highlighting treatable and novel genetic causes. Clin. Genet..

[24] Chen L., Cui Y., Jiang D., Ma C.Y., Tse H.-F., Hwu W.-L., Lian Q. (2018). Management of Leigh syndrome: Current status and new insights. Clin. Genet..

[25] Chang X., Wu Y., Zhou J., Meng H., Zhang W., Guo J. (2020). A meta-analysis and systematic review of Leigh syndrome: Clinical manifestations, respiratory chain enzyme complex deficiency, and gene mutations. Medicine.

[26] Ball M., Thorburn D.R., Rahman S., Adam M.P., Feldman J., Mirzaa G.M., Pagon R.A., Wallace S.E., Amemiya A. (2003). Mitochondrial DNA-Associated Leigh Syndrome Spectrum. GeneReviews^®^.

[27] Yang Y.L., Sun F., Zhang Y., Qian N., Yuan Y., Wang Z.X., Qi Y., Xiao J.X., Wang X.Y., Qi Z.Y. (2006). Clinical and laboratory survey of 65 Chinese patients with Leigh syndrome. Chin. Med. J..

[28] Bugiani M., Tiranti V., Farina L., Uziel G., Zeviani M. (2005). Novel mutations in COX15 in a long surviving Leigh syndrome patient with cytochrome c oxidase deficiency. J. Med. Genet..

[29] Desguerre I., Pinton F., Nabbout R., Moutard M.L., N’Guyen S., Marsac C., Ponsot G., Dulac O. (2003). Infantile spasms with basal ganglia MRI hypersignal may reveal mitochondrial disorder due to T8993G MT DNA mutation. Neuropediatrics.

[30] Pequignot M.O., Desguerre I., Dey R., Tartari M., Zeviani M., Agostino A., Benelli C., Fouque F., Prip-Buus C., Marchant D. (2001). New splicing-site mutations in the SURF1 gene in Leigh syndrome patients. J. Biol. Chem..

[31] Mak S.C., Chi C.S., Tsai C.R. (1998). Mitochondrial DNA 8993 T > C mutation presenting as juvenile Leigh syndrome with respiratory failure. J. Child Neurol..

[32] Sofou K., De Coo I.F.M., Isohanni P., Ostergaard E., Naess K., De Meirleir L., Tzoulis C., Uusimaa J., De Angst I.B., Lönnqvist T. (2014). A multicenter study on Leigh syndrome: Disease course and predictors of survival. Orphanet J. Rare Dis..

[33] Lee S., Na J.H., Lee Y.M. (2019). Epilepsy in Leigh Syndrome With Mitochondrial DNA Mutations. Front. Neurol..

[34] Ng Y.S., Martikainen M.H., Gorman G.S., Blain A., Bugiardini E., Bunting A., Schaefer A.M., Alston C.L., Blakely E.L., Sharma S. (2019). Pathogenic variants in MT-ATP6: A United Kingdom-based mitochondrial disease cohort study. Ann. Neurol..

[35] Macaya A., Munell F., Burke R.E., De Vivo D.C. (1993). Disorders of movement in Leigh syndrome. Neuropediatrics.

[36] Tranchant C., Anheim M. (2016). Movement disorders in mitochondrial diseases. Rev. Neurol..

[37] Alston C.L., Morak M., Reid C., Hargreaves I.P., Pope S.A.S., Land J.M., Heales S.J., Horvath R., Mundy H., Taylor R.W. (2010). A novel mitochondrial MTND5 frameshift mutation causing isolated complex I deficiency, renal failure and myopathy. Neuromuscul. Disord..

[38] Farina L., Chiapparini L., Uziel G., Bugiani M., Zeviani M., Savoiardo M. (2002). MR findings in Leigh syndrome with COX deficiency and SURF-1 mutations. Am. J. Neuroradiol..

[39] Sofou K., Steneryd K., Wiklund L.M., Tulinius M., Darin N. (2013). MRI of the brain in childhood-onset mitochondrial disorders with central nervous system involvement. Mitochondrion.

[40] Stenton S.L., Zou Y., Cheng H., Liu Z., Wang J., Shen D., Jin H., Ding C., Tang X., Sun S. (2022). Leigh Syndrome: A Study of 209 Patients at the Beijing Children’s Hospital. Ann. Neurol..

[41] Kistol D., Tsygankova P., Krylova T., Bychkov I., Itkis Y., Nikolaeva E., Mikhailova S., Sumina M., Pechatnikova N., Kurbatov S. (2023). Leigh Syndrome: Spectrum of Molecular Defects and Clinical Features in Russia. Int. J. Mol. Sci..

[42] Rahman S. (2013). Gastrointestinal and hepatic manifestations of mitochondrial disorders. J. Inherit. Metab. Dis..

[43] Lim A.Z., Ng Y.S., Blain A., Jiminez-Moreno C., Alston C.L., Nesbitt V., Simmons L., Santra S., Wassmer E., Blakely E.L. (2022). Natural History of Leigh Syndrome: A Study of Disease Burden and Progression. Ann. Neurol..

[44] Naess K., Freyer C., Bruhn H., Wibom R., Malm G., Nennesmo I., von Döbeln U., Larsson N.G. (2009). MtDNA mutations are a common cause of severe disease phenotypes in children with Leigh syndrome. Biochim. Biophys. Acta.

[45] Van Hove J.L., Saenz M.S., Thomas J.A., Gallagher R.C., Lovell M.A., Fenton L.Z., Shanske S., Myers S.M., Wanders R.J., Ruiter J. (2010). Succinyl-CoA ligase deficiency: A mitochondrial hepatoencephalomyopathy. Pediatr. Res..

[46] López L.C., Schuelke M., Quinzii C.M., Kanki T., Rodenburg R.J., Naini A., Dimauro S., Hirano M. (2006). Leigh syndrome with nephropathy and CoQ10 deficiency due to decaprenyl diphosphate synthase subunit 2 (PDSS2) mutations. Am. J. Hum. Genet..

[47] Sofou K., de Coo I.F.M., Ostergaard E., Isohanni P., Naess K., De Meirleir L., Tzoulis C., Uusimaa J., Lönnqvist T., Bindoff L.A. (2018). Phenotype-genotype correlations in Leigh syndrome: New insights from a multicentre study of 96 patients. J. Med. Genet..

[48] Sonam K., Khan N.A., Bindu P.S., Taly A.B., Gayathri N., Bharath M.M.S., Govindaraju C., Arvinda H.R., Nagappa M., Sinha S. (2014). Clinical and magnetic resonance imaging findings in patients with Leigh syndrome and SURF1 mutations. Brain Dev..

[49] Østergaard E., Bradinova I., Ravn S.H., Hansen F.J., Simeonov E., Christensen E., Wibrand F., Schwartz M. (2005). Hypertrichosis in patients with SURF1 mutations. Am. J. Med. Genet. Part A.

[50] Gerards M., Sallevelt S.C.E.H., Smeets H.J.M. (2016). Leigh syndrome: Resolving the clinical and genetic heterogeneity paves the way for treatment options. Mol. Genet. Metab..

[51] Zhang Y., Yang Y.L., Sun F., Cai X., Qian N., Yuan Y., Wang Z.X., Qi Y., Xiao J.X., Wang X.Y. (2007). Clinical and molecular survey in 124 Chinese patients with Leigh or Leigh-like syndrome. J. Inherit. Metab. Dis..

[52] Munaro M., Tiranti V., Sandonà D., Lamantea E., Uziel G., Bisson R., Zeviani M. (1997). A single cell complementation class is common to several cases of cytochrome c oxidase- defective Leigh’s syndrome. Hum. Mol. Genet..

[53] Nagashima T., Mori M., Katayama K., Nunomura M., Nishihara H., Hiraga H., Tanaka S., Goto Y., Nagashima K. (1999). Adult Leigh syndrome with mitochondrial DNA mutation at 8993. Acta Neuropathol..

[54] Debray F.G., Lambert M., Chevalier I., Robitaille Y., Decarie J.C., Shoubridge E.A., Robinson B.H., Mitchell G.A. (2007). Long-term outcome and clinical spectrum of 73 pediatric patients with mitochondrial diseases. Pediatrics.

[55] Van Maldergem L., Trijbels F., DiMauro S., Sindelar P.J., Musumeci O., Janssen A., Delberghe X., Martin J.J., Gillerot Y. (2002). Coenzyme Q-responsive Leigh’s encephalopathy in two sisters. Ann. Neurol..

[56] Malandrini A., Palmeri S., Fabrizi G.M., Villanova M., Berti G., Salvadori C., Gardini G., Motti L., Solimé F., Guazzi G.C. (1998). Juvenile Leigh syndrome with protracted course presenting as chronic sensory motor neuropathy, ataxia, deafness and retinitis pigmentosa: A clinicopathological report. J. Neurol. Sci..

[57] Alves C.A.P.F., Teixeira S.R., Martin-Saavedra J.S., Guimarães Gonçalves F., Lo Russo F., Muraresku C., McCormick E.M., Falk M.J., Zolkipli-Cunningham Z., Ganetzky R. (2020). Pediatric Leigh Syndrome: Neuroimaging Features and Genetic Correlations. Ann. Neurol..

[58] Van de Wal M.A.E., Adjobo-Hermans M.J.W., Keijer J., Schirris T.J.J., Homberg J.R., Wieckowski M.R., Grefte S., van Schothorst E.M., van Karnebeek C., Quintana A. (2022). Ndufs4 knockout mouse models of Leigh syndrome: Pathophysiology and intervention. Brain.

[59] Calvaruso M.A., Willems P., van den Brand M., Valsecchi F., Kruse S., Palmiter R., Smeitink J., Nijtmans L. (2012). Mitochondrial complex III stabilizes complex I in the absence of NDUFS4 to provide partial activity. Hum. Mol. Genet..

[60] Liu L., Zhang K., Sandoval H., Yamamoto S., Jaiswal M., Sanz E., Li Z., Hui J., Graham B.H., Quintana A. (2015). Glial lipid droplets and ROS induced by mitochondrial defects promote neurodegeneration. Cell.

[61] Procaccio V., Wallace D.C. (2004). Late-onset Leigh syndrome in a patient with mitochondrial complex I NDUFS8 mutations. Neurology.

[62] Wang S., Kang Y., Wang R., Deng J., Yu Y., Yu J., Wang J. (2022). Emerging Roles of NDUFS8 Located in Mitochondrial Complex I in Different Diseases. Molecules.

[63] Kirby D.M., Kahler S.G., Freckmann M.L., Reddihough D., Thorburn D.R. (2000). Leigh disease caused by the mitochondrial DNA G14459A mutation in unrelated families. Ann. Neurol..

[64] Nesbitt V., Morrison P.J., Crushell E., Donnelly D.E., Alston C.L., He L., McFarland R., Taylor R.W. (2012). The clinical spectrum of the m.10191T>C mutation in complex I-deficient Leigh syndrome. Dev. Med. Child Neurol..

[65] Rustin P., Rötig A. (2002). Inborn errors of complex II—Unusual human mitochondrial diseases. Biochim. Biophys. Acta.

[66] Alston C.L., Davison J.E., Meloni F., van der Westhuizen F.H., He L., Hornig-Do H.T., Peet A.C., Gissen P., Goffrini P., Ferrero I. (2012). Recessive germline SDHA and SDHB mutations causing leukodystrophy and isolated mitochondrial complex II deficiency. J. Med. Genet..

[67] Brockmann K., Bjornstad A., Dechent P., Korenke C.G., Smeitink J., Trijbels J.M.F., Athanassopoulos S., Villagran R., Skjeldal O.H., Wilichowski E. (2002). Succinate in dystrophic white matter: A proton magnetic resonance spectroscopy finding characteristic for complex II deficiency. Ann. Neurol..

[68] Atwal P.S. (2014). Mutations in the Complex III Assembly Factor Tetratricopeptide 19 Gene TTC19 Are a Rare Cause of Leigh Syndrome. JIMD Rep..

[69] Koch J., Feichtinger R.G., Freisinger P., Pies M., Schrödl F., Iuso A., Sperl W., Mayr J.A., Prokisch H., Haack T.B. (2016). Disturbed mitochondrial and peroxisomal dynamics due to loss of MFF causes Leigh-like encephalopathy, optic atrophy and peripheral neuropathy. J. Med. Genet..

[70] Spinazzi M., Radaelli E., Horré K., Arranz A.M., Gounko N.V., Agostinis P., Maia T.M., Impens F., Morais V.A., Lopez-Lluch G. (2019). PARL deficiency in mouse causes Complex III defects, coenzyme Q depletion, and Leigh-like syndrome. Proc. Natl. Acad. Sci. USA.

[71] Barel O., Shorer Z., Flusser H., Ofir R., Narkis G., Finer G., Shalev H., Nasasra A., Saada A., Birk O.S. (2008). Mitochondrial Complex III Deficiency Associated with a Homozygous Mutation in UQCRQ. Am. J. Hum. Genet..

[72] Misceo D., Strømme P., Bitarafan F., Chawla M.S., Sheng Y., Bach de Courtade S.M., Eide L., Frengen E. (2024). Biallelic NDUFA4 Deletion Causes Mitochondrial Complex IV Deficiency in a Patient with Leigh Syndrome. Genes.

[73] Lee I.C., Chiang K.L. (2021). Clinical Diagnosis and Treatment of Leigh Syndrome Based on SURF1: Genotype and Phenotype. Antioxidants.

[74] Wedatilake Y., Brown R.M., McFarland R., Yaplito-Lee J., Morris A.A., Champion M., Jardine P.E., Clarke A., Thorburn D.R., Taylor R.W. (2013). SURF1 deficiency: A multi-centre natural history study. Orphanet J. Rare Dis..

[75] Bartke A. (2008). New findings in gene knockout, mutant and transgenic mice. Exp. Gerontol..

[76] Thorburn D.R., Rahman J., Rahman S. (1993). Mitochondrial DNA-Associated Leigh Syndrome and NARP.

[77] Ganapathi M., Friocourt G., Gueguen N., Friederich M.W., Le Gac G., Okur V., Loaëc N., Ludwig T., Ka C., Tanji K. (2022). A homozygous splice variant in ATP5PO, disrupts mitochondrial complex V function and causes Leigh syndrome in two unrelated families. J. Inherit. Metab. Dis..

[78] Patel K.P., O’Brien T.W., Subramony S.H., Shuster J., Stacpoole P.W. (2012). The spectrum of pyruvate dehydrogenase complex deficiency: Clinical, biochemical and genetic features in 371 patients. Mol. Genet. Metab..

[79] Quinonez S.C., Thoene J.G. (1993). Dihydrolipoamide Dehydrogenase Deficiency.

[80] Mitchell G., Ogier H., Munnich A., Saudubray J.M., Shirrer J., Charpentier C., Rocchiccioli F. (1986). Neurological Deterioration and Lactic Acidemia in Biotinidase Deficiency. A Treatable Cond. Mimicking Leigh’s Dis..

[81] Gerards M., Kamps R., van Oevelen J., Boesten I., Jongen E., de Koning B., Scholte H.R., de Angst I., Schoonderwoerd K., Sefiani A. (2013). Exome sequencing reveals a novel Moroccan founder mutation in SLC19A3 as a new cause of early-childhood fatal Leigh syndrome. Brain.

[82] Fassone E., Rahman S. (2012). Complex I deficiency: Clinical features, biochemistry and molecular genetics. J. Med. Genet..

[83] Carrozzo R., Verrigni D., Rasmussen M., de Coo R., Amartino H., Bianchi M., Buhas D., Mesli S., Naess K., Born A.P. (2016). Succinate-CoA ligase deficiency due to mutations in SUCLA2 and SUCLG1: Phenotype and genotype correlations in 71 patients. J. Inherit. Metab. Dis..

[84] Wortmann S.B., van Hasselt P.M., Barić I., Burlina A., Darin N., Hörster F., Coker M., Ucar S.K., Krumina Z., Naess K. (2015). Eyes on MEGDEL: Distinctive basal ganglia involvement in dystonia deafness syndrome. Neuropediatrics.

[85] Bénit P., Chretien D., Kadhom N., de Lonlay-Debeney P., Cormier-Daire V., Cabral A., Peudenier S., Rustin P., Munnich A., Rötig A. (2001). Large-scale deletion and point mutations of the nuclear NDUFV1 and NDUFS1 genes in mitochondrial complex I deficiency. Am. J. Hum. Genet..

[86] Horváth R., Abicht A., Holinski-Feder E., Laner A., Gempel K., Prokisch H., Lochmüller H., Klopstock T., Jaksch M. (2006). Leigh syndrome caused by mutations in the flavoprotein (Fp) subunit of succinate dehydrogenase (SDHA). J. Neurol. Neurosurg. Psychiatry.

[87] Lebon S., Chol M., Benit P., Mugnier C., Chretien D., Giurgea I., Kern I., Girardin E., Hertz-Pannier L., de Lonlay P. (2003). Recurrent de novo mitochondrial DNA mutations in respiratory chain deficiency. J. Med. Genet..

[88] Wortmann S., Rodenburg R.J., Huizing M., Loupatty F.J., de Koning T., Kluijtmans L.A., Engelke U., Wevers R., Smeitink J.A., Morava E. (2006). Association of 3-methylglutaconic aciduria with sensori-neural deafness, encephalopathy, and Leigh-like syndrome (MEGDEL association) in four patients with a disorder of the oxidative phosphorylation. Mol. Genet. Metab..

[89] Martín M.A., Blázquez A., Gutierrez-Solana L.G., Fernández-Moreira D., Briones P., Andreu A.L., Garesse R., Campos Y., Arenas J. (2005). Leigh syndrome associated with mitochondrial complex I deficiency due to a novel mutation in the NDUFS1 gene. Arch. Neurol..

[90] Malfatti E., Bugiani M., Invernizzi F., de Souza C.F., Farina L., Carrara F., Lamantea E., Antozzi C., Confalonieri P., Sanseverino M.T. (2007). Novel mutations of ND genes in complex I deficiency associated with mitochondrial encephalopathy. Brain.

[91] Rossi A., Biancheri R., Bruno C., Di Rocco M., Calvi A., Pessagno A., Tortori-Donati P. (2003). Leigh Syndrome with COX deficiency and SURF1 gene mutations: MR imaging findings. Am. J. Neuroradiol..

[92] Loeffen J., Smeitink J., Triepels R., Smeets R., Schuelke M., Sengers R., Trijbels F., Hamel B., Mullaart R., van den Heuvel L. (1998). The first nuclear-encoded complex I mutation in a patient with Leigh syndrome. Am. J. Hum. Genet..

[93] Araki S., Hayashi M., Yasaka A., Maruki K. (1997). Electrophysiological brainstem dysfunction in a child with Leigh disease. Pediatr. Neurol..

[94] Martin E., Burger R., Wiestler O.D., Caduff R., Boltshauser E., Boesch C. (1990). Brainstem lesion revealed by MRI in a case of Leigh’s disease with respiratory failure. Pediatr. Radiol..

[95] Bonfante E., Koenig M.K., Adejumo R.B., Perinjelil V., Riascos R.F. (2016). The neuroimaging of Leigh syndrome: Case series and review of the literature. Pediatr. Radiol..

[96] Rio M., Lebre A.S., de Lonlay P., Valayannopoulos V., Desguerre I., Dufier J.L., Grévent D., Zilbovicius M., Tréguier C., Brunelle F. (2010). Mitochondrial ND5 mutations mimicking brainstem tectal glioma. Neurology.

[97] Bindu P.S., Taly A.B., Sonam K., Govindaraju C., Arvinda H.R., Gayathri N., Bharath M.M., Ranjith D., Nagappa M., Sinha S. (2014). Bilateral hypertrophic olivary nucleus degeneration on magnetic resonance imaging in children with Leigh and Leigh-like syndrome. Br. J. Radiol..

[98] Rahman S., Thorbur D. (2020). Nuclear Gene-Encoded Leigh Syndrome Spectrum Overview. Gene Reviews.

[99] Tiet M.Y., Lin Z., Gao F., Jennings M.J., Horvath R. (2021). Targeted Therapies for Leigh Syndrome: Systematic Review and Steps Towards a ‘Treatabolome’. J. Neuromuscul. Dis..

[100] Montini G., Malaventura C., Salviati L. (2008). Early coenzyme Q10 supplementation in primary coenzyme Q10 deficiency. N. Engl. J. Med..

[101] Chen Z., Zhao Z., Ye Q., Chen Y., Pan X., Sun B., Huang H., Zheng A. (2015). Mild clinical manifestation and unusual recovery upon coenzyme Q_10_ treatment in the first Chinese Leigh syndrome pedigree with mutation m.10197 G > A. Mol. Med. Rep..

[102] Scalais E., Chafai R., Van Coster R., Bindl L., Nuttin C., Panagiotaraki C., Seneca S., Lissens W., Ribes A., Geers C. (2013). Early myoclonic epilepsy, hypertrophic cardiomyopathy and subsequently a nephrotic syndrome in a patient with CoQ10 deficiency caused by mutations in para-hydroxybenzoate-polyprenyl transferase (COQ2). Eur. J. Paediatr. Neurol..

[103] Haginoya K., Miyabayashi S., Kikuchi M., Kojima A., Yamamoto K., Omura K., Uematsu M., Hino-Fukuyo N., Tanaka S., Tsuchiya S. (2009). Efficacy of idebenone for respiratory failure in a patient with Leigh syndrome: A long-term follow-up study. J. Neurol. Sci..

[104] Enns G.M., Kinsman S.L., Perlman S.L., Spicer K.M., Abdenur J.E., Cohen B.H., Amagata A., Barnes A., Kheifets V., Shrader W.D. (2012). Initial experience in the treatment of inherited mitochondrial disease with EPI-743. Mol. Genet. Metab..

[105] Martinelli D., Catteruccia M., Piemonte F., Pastore A., Tozzi G., Dionisi-Vici C., Pontrelli G., Corsetti T., Livadiotti S., Kheifets V. (2012). EPI-743 reverses the progression of the pediatric mitochondrial disease—Genetically defined Leigh Syndrome. Mol. Genet. Metab..

[106] Fujii T., Ito M., Miyajima T., Okuno T. (2002). Dichloroacetate therapy in Leigh syndrome with a mitochondrial T8993C mutation. Pediatr. Neurol..

[107] Kimura S., Osaka H., Saitou K., Ohtuki N., Kobayashi T., Nezu A. (1995). Improvement of lesions shown on MRI and CT scan by administration of dichloroacetate in patients with Leigh syndrome. J. Neurol. Sci..

[108] Spruijt L., Naviaux R.K., McGowan K.A., Nyhan W.L., Sheean G., Haas R.H., Barshop B.A. (2001). Nerve conduction changes in patients with mitochondrial diseases treated with dichloroacetate. Muscle Nerve.

[109] Koga Y., Povalko N., Katayama K., Kakimoto N., Matsuishi T., Naito E., Tanaka M. (2012). Beneficial effect of pyruvate therapy on Leigh syndrome due to a novel mutation in PDH E1α gene. Brain Dev..

[110] Tanaka M., Nishigaki Y., Fuku N., Ibi T., Sahashi K., Koga Y. (2007). Therapeutic potential of pyruvate therapy for mitochondrial diseases. Mitochondrion.

[111] Komaki H., Nishigaki Y., Fuku N., Hosoya H., Murayama K., Ohtake A., Goto Y., Wakamoto H., Koga Y., Tanaka M. (2010). Pyruvate therapy for Leigh syndrome due to cytochrome c oxidase deficiency. Biochim. Biophys. Acta.

[112] Fujii T., Nozaki F., Saito K., Hayashi A., Nishigaki Y., Murayama K., Tanaka M., Koga Y., Hiejima I., Kumada T. (2014). Efficacy of pyruvate therapy in patients with mitochondrial disease: A semi-quantitative clinical evaluation study. Mol. Genet. Metab..

[113] Koene S., Spaans E., Van Bortel L., Van Lancker G., Delafontaine B., Badilini F., Beyrath J., Smeitink J. (2017). KH176 under development for rare mitochondrial disease: A first in man randomized controlled clinical trial in healthy male volunteers. Orphanet J. Rare Dis..

[114] Janssen M.C.H., Koene S., de Laat P., Hemelaar P., Pickkers P., Spaans E., Beukema R., Beyrath J., Groothuis J., Verhaak C. (2019). The KHENERGY Study: Safety and Efficacy of KH176 in Mitochondrial m.3243A>G Spectrum Disorders. Clin. Pharmacol. Ther..

[115] Garone C., Donati M.A., Sacchini M., Garcia-Diaz B., Bruno C., Calvo S., Mootha V.K., Dimauro S. (2013). Mitochondrial encephalomyopathy due to a novel mutation in ACAD9. JAMA Neurol..

[116] Gerards M., van den Bosch B.J., Danhauser K., Serre V., van Weeghel M., Wanders R.J., Nicolaes G.A., Sluiter W., Schoonderwoerd K., Scholte H.R. (2011). Riboflavin-responsive oxidative phosphorylation complex I deficiency caused by defective ACAD9: New function for an old gene. Brain.

[117] Ortigoza-Escobar J.D., Molero-Luis M., Arias A., Oyarzabal A., Darín N., Serrano M., Garcia-Cazorla A., Tondo M., Hernández M., Garcia-Villoria J. (2016). Free-thiamine is a potential biomarker of thiamine transporter-2 deficiency: A treatable cause of Leigh syndrome. Brain.

[118] Haack T.B., Klee D., Strom T.M., Mayatepek E., Meitinger T., Prokisch H., Distelmaier F. (2014). Infantile Leigh-like syndrome caused by SLC19A3 mutations is a treatable disease. Brain.

[119] Debs R., Depienne C., Rastetter A., Bellanger A., Degos B., Galanaud D., Keren B., Lyon-Caen O., Brice A., Sedel F. (2010). Biotin-responsive basal ganglia disease in ethnic Europeans with novel SLC19A3 mutations. Arch. Neurol..

[120] Tabarki B., Alfadhel M., AlShahwan S., Hundallah K., AlShafi S., AlHashem A. (2015). Treatment of biotin-responsive basal ganglia disease: Open comparative study between the combination of biotin plus thiamine versus thiamine alone. Eur. J. Paediatr. Neurol..

[121] Bottani E., Lamperti C., Prigione A., Tiranti V., Persico N., Brunetti D. (2020). Therapeutic Approaches to Treat Mitochondrial Diseases: “One-Size-Fits-All” and “Precision Medicine” Strategies. Pharmaceutics.

[122] Viscomi C., Burlina A.B., Dweikat I., Savoiardo M., Lamperti C., Hildebrandt T., Tiranti V., Zeviani M. (2010). Combined treatment with oral metronidazole and N-acetylcysteine is effective in ethylmalonic encephalopathy. Nat. Med..

[123] Shayota B.J., Soler-Alfonso C., Bekheirnia M.R., Mizerik E., Boyer S.W., Xiao R., Yang Y., Elsea S.H., Scaglia F. (2019). Case report and novel treatment of an autosomal recessive Leigh syndrome caused by short-chain enoyl-CoA hydratase deficiency. Am. J. Med. Genet. A.

[124] Tarnopolsky M.A. (2008). The mitochondrial cocktail: Rationale for combined nutraceutical therapy in mitochondrial cytopathies. Adv. Drug Deliv. Rev..

[125] Tóth G., Morava E., Bene J., Selhorst J.J., Overmars H., Vreken P., Molnár J., Farkas V., Melegh B. (2001). Carnitine-responsive carnitine insufficiency in a case of mtDNA 8993T > C mutation associated Leigh syndrome. J. Inherit. Metab. Dis..

[126] Wexler I.D., Hemalatha S.G., McConnell J., Buist N.R., Dahl H.H., Berry S.A., Cederbaum S.D., Patel M.S., Kerr D.S. (1997). Outcome of pyruvate dehydrogenase deficiency treated with ketogenic diets. Studies in patients with identical mutations. Neurology.

[127] Laugel V., This-Bernd V., Cormier-Daire V., Speeg-Schatz C., de Saint-Martin A., Fischbach M. (2007). Early-onset ophthalmoplegia in Leigh-like syndrome due to NDUFV1 mutations. Pediatr. Neurol..

[128] Banka S., de Goede C., Yue W.W., Morris A.A., von Bremen B., Chandler K.E., Feichtinger R.G., Hart C., Khan N., Lunzer V. (2014). Expanding the clinical and molecular spectrum of thiamine pyrophosphokinase deficiency: A treatable neurological disorder caused by TPK1 mutations. Mol. Genet. Metab..

[129] Reardon S. Three-Parent Baby; Claim Raises Hopes and Ethical Concerns. http://www.nature.com/news/three-parent-baby-claim-raises-hopes-and-ethical-concerns-1.

[130] Zhang J., Liu H., Luo S., Lu Z., Chávez-Badiola A., Liu Z., Yang M., Merhi Z., Silber S.J., Munné S. (2017). Live birth derived from oocyte spindle transfer to prevent mitochondrial disease. Reprod. Biomed. Online.

[131] Quintana A., Zanella S., Koch H., Kruse S.E., Lee D., Ramirez J.M., Palmiter R.D. (2012). Fatal breathing dysfunction in a mouse model of Leigh syndrome. J. Clin. Investig..

[132] Hanaford A.R., Cho Y.J., Nakai H. (2022). AAV-vector based gene therapy for mitochondrial disease: Progress and future perspectives. Orphanet J. Rare Dis..

[133] Nakai R., Varnum S., Field R.L., Shi H., Giwa R., Jia W., Krysa S.J., Cohen E.F., Borcherding N., Saneto R.P. (2024). Mitochondria transfer-based therapies reduce the morbidity and mortality of Leigh syndrome. Nat. Metab..

[134] Henke M.T., Prigione A., Schuelke M. (2024). Disease models of Leigh syndrome: From yeast to organoids. J. Inherit. Metab. Dis..

[135] Lee C.F., Caudal A., Abell L., Nagana Gowda G.A., Tian R. (2019). Targeting NAD+ Metabolism as Interventions for Mitochondrial Disease. Sci. Rep..

[136] Johnson S.C., Yanos M.E., Kayser E.B., Quintana A., Sangesland M., Castanza A., Uhde L., Hui J., Wall V.Z., Gagnidze A. (2013). mTOR Inhibition Alleviates Mitochondrial Disease in a Mouse Model of Leigh Syndrome. Science.

[137] Felici R., Cavone L., Lapucci A., Guasti D., Bani D., Chiarugi A. (2014). PARP Inhibition Delays Progression of Mitochondrial Encephalopathy in Mice. Neurotherapeutics.

[138] Martin-Perez M., Grillo A.S., Ito T.K., Valente A.S., Han J., Entwisle S.W., Huang H.Z., Kim D., Yajima M., Kaeberlein M. (2020). PKC downregulation upon rapamycin treatment attenuates mitochondrial disease. Nat. Metab..

[139] Jain I.H., Zazzeron L., Goli R., Alexa K., Schatzman-Bone S., Dhillon H., Goldberger O., Peng J., Shalem O., Sanjana N.E. (2016). Hypoxia as a therapy for mitochondrial disease. Science.

[140] Ferrari M., Jain I.H., Goldberger O., Rezoagli E., Thoonen R., Cheng K.-H., Sosnovik D.E., Scherrer-Crosbie M., Mootha V.K., Zapol W.M. (2017). Hypoxia treatment reverses neurodegenerative disease in a mouse model of Leigh syndrome. Proc. Natl. Acad. Sci. USA.

[141] Walker M.A., Miranda M., Allred A., Mootha V.K. (2022). On the dynamic and even reversible nature of Leigh syndrome: Lessons from human imaging and mouse models. Curr. Opin. Neurobiol..

[142] Jain I.H., Zazzeron L., Goldberger O., Marutani E., Wojtkiewicz G.R., Ast T., Wang H., Schleifer G., Stepanova A., Brepoels K. (2019). Leigh Syndrome Mouse Model Can Be Rescued by Interventions that Normalize Brain Hyperoxia, but Not HIF Activation. Cell Metab..

[143] Barrientos A., Korr D., Tzagoloff A. (2002). Shy1p is necessary for full expression of mitochondrial COX1 in the yeast model of Leigh’s syndrome. Embo J..

[144] Haroon S., Yoon H., Seiler C., Osei-Frimpong B., He J., Nair R.M., Mathew N.D., Burg L., Kose M., Venkata C.R.M. (2023). N-acetylcysteine and cysteamine bitartrate prevent azide-induced neuromuscular decompensation by restoring glutathione balance in two novel surf1-/- zebrafish deletion models of Leigh syndrome. Hum. Mol. Genet..

[145] Burman J.L., Itsara L.S., Kayser E.B., Suthammarak W., Wang A.M., Kaeberlein M., Sedensky M.M., Morgan P.G., Pallanck L.J. (2014). A Drosophila model of mitochondrial disease caused by a complex I mutation that uncouples proton pumping from electron transfer. Dis. Model. Mech..

[146] Wang A., Mouser J., Pitt J., Promislow D., Kaeberlein M. (2016). Rapamycin enhances survival in a Drosophila model of mitochondrial disease. Oncotarget.

[147] Olufs Z.P.G., Ganetzky B., Wassarman D.A., Perouansky M. (2020). Mitochondrial Complex I Mutations Predispose Drosophila to Isoflurane Neurotoxicity. Anesthesiology.

[148] Borchardt L.A., Scharenbrock A.R., Olufs Z.P.G., Wassarman D.A., Perouansky M. (2023). Mutations in Complex I of the Mitochondrial Electron-Transport Chain Sensitize the Fruit Fly (*Drosophila melanogaster*) to Ether and Non-Ether Volatile Anesthetics. Int. J. Mol. Sci..

[149] Wojtala A., Karkucinska-Wieckowska A., Sardao V.A., Szczepanowska J., Kowalski P., Pronicki M., Duszynski J., Wieckowski M.R. (2017). Modulation of mitochondrial dysfunction-related oxidative stress in fibroblasts of patients with Leigh syndrome by inhibition of prooxidative p66Shc pathway. Mitochondrion.

[150] Takahashi K., Yamanaka S. (2006). Induction of pluripotent stem cells from mouse embryonic and adult fibroblast cultures by defined factors. Cell.

[151] Lorenz C., Lesimple P., Bukowiecki R., Zink A., Inak G., Mlody B., Singh M., Semtner M., Mah N., Auré K. (2017). Human iPSC-Derived Neural Progenitors Are an Effective Drug Discovery Model for Neurological mtDNA Disorders. Cell Stem Cell.

[152] Prigione A., Viscomi C. (2022). How to mitigate neurological and cardiac decompensation in Leigh syndrome: Can nicotinamide riboside be an answer?. Clin. Transl. Discov..

[153] Bieganowski P., Brenner C. (2004). Discoveries of nicotinamide riboside as a nutrient and conserved NRK genes establish a Preiss-Handler independent route to NAD+ in fungi and humans. Cell.

[154] Price N.L., Gomes A.P., Ling A.J., Duarte F.V., Martin-Montalvo A., North B.J., Agarwal B., Ye L., Ramadori G., Teodoro J.S. (2012). SIRT1 is required for AMPK activation and the beneficial effects of resveratrol on mitochondrial function. Cell Metab..

[155] Rodgers J.T., Lerin C., Haas W., Gygi S.P., Spiegelman B.M., Puigserver P. (2005). Nutrient control of glucose homeostasis through a complex of PGC-1alpha and SIRT1. Nature.

[156] Bai P., Cantó C., Oudart H., Brunyánszki A., Cen Y., Thomas C., Yamamoto H., Huber A., Kiss B., Houtkooper R.H. (2011). PARP-1 inhibition increases mitochondrial metabolism through SIRT1 activation. Cell Metab..

[157] Cantó C., Menzies K.J., Auwerx J. (2015). NAD_+_ Metabolism and the Control of Energy Homeostasis: A Balancing Act between Mitochondria and the Nucleus. Cell Metab..

[158] Li J., Kim S.G., Blenis J. (2014). Rapamycin: One drug, many effects. Cell Metab..

[159] Johnson S.C. (2014). Translational Medicine. A target for pharmacological intervention in an untreatable human disease. Science.

[160] Geach T. (2014). Neurometabolic disease: Treating mitochondrial diseases with mTOR inhibitors—A potential treatment for Leigh syndrome?. Nat. Rev. Neurol..

[161] Puighermanal E., Luna-Sánchez M., Gella A., van der Walt G., Urpi A., Royo M., Tena-Morraja P., Appiah I., de Donato M.H., Menardy F. (2024). Cannabidiol ameliorates mitochondrial disease via PPARγ activation in preclinical models. Nat. Commun..

[162] Ling Q., Rioux M., Hu Y., Lee M., Gray S.J. (2021). Adeno-associated viral vector serotype 9-based gene replacement therapy for SURF1-related Leigh syndrome. Mol. Ther. Methods Clin. Dev..

